# The Landscape of Recombination Events That Create Nonribosomal Peptide Diversity

**DOI:** 10.1093/molbev/msab015

**Published:** 2021-01-22

**Authors:** Martin Baunach, Somak Chowdhury, Pierre Stallforth, Elke Dittmann

**Affiliations:** 1 Institute for Biochemistry and Biology, University of Potsdam, Potsdam-Golm, Germany; 2 Department of Paleobiotechnology, Leibniz Institute for Natural Product Research and Infection Biology – Hans Knöll Institute (HKI), Jena, Germany

**Keywords:** evolution, recombination, structural diversity, natural products, nonribosomal peptide synthetases, microbial ecology

## Abstract

Nonribosomal peptides (NRP) are crucial molecular mediators in microbial ecology and provide indispensable drugs. Nevertheless, the evolution of the flexible biosynthetic machineries that correlates with the stunning structural diversity of NRPs is poorly understood. Here, we show that recombination is a key driver in the evolution of bacterial NRP synthetase (NRPS) genes across distant bacterial phyla, which has guided structural diversification in a plethora of NRP families by extensive mixing and matching of biosynthesis genes. The systematic dissection of a large number of individual recombination events did not only unveil a striking plurality in the nature and origin of the exchange units but allowed the deduction of overarching principles that enable the efficient exchange of adenylation (A) domain substrates while keeping the functionality of the dynamic multienzyme complexes. In the majority of cases, recombination events have targeted variable portions of the A_core_ domains, yet domain interfaces and the flexible A_sub_ domain remained untapped. Our results strongly contradict the widespread assumption that adenylation and condensation (C) domains coevolve and significantly challenge the attributed role of C domains as stringent selectivity filter during NRP synthesis. Moreover, they teach valuable lessons on the choice of natural exchange units in the evolution of NRPS diversity, which may guide future engineering approaches.

## Introduction

Nonribosomal peptides (NRPs) are one of the most diverse and widespread classes of natural products. They are of tremendous importance in microbial ecology as virulence factors and toxins among others such as the siderophore mycobactin. In human health, they serve as life-saving drugs that greatly contribute to human welfare as showcased by the antibiotic vancomycin or the immunosuppressant cyclosporine A (Süssmuth and Mainz 2017). Biochemical and structural studies have greatly enhanced our mechanistic understanding of the underlying biosynthesis enzymes, assembly line-like megasynthetases (Drake et al. 2016; Reimer et al. 2016, 2019; Süssmuth and Mainz 2017). Strikingly, the evolution of the vast diversity of individual megasynthetases that correlates with the stunning structural diversity and complexity of this indispensable class of compounds is poorly understood (Chevrette et al. 2020).

While in ribosomal peptide synthesis the sequence of the final peptide is encoded in the DNA and can be universally translated by the ribosome, nonribosomal peptide synthetases (NRPS) are customized for the synthesis of restricted sets of related compounds (Brown et al. 2018). These enzymes have a modular architecture and follow an assembly line logic in which individual modules are responsible for the selection, activation, processing, and connection of a specific amino acid to a further one. Modules consist of adenylation (A) domains for amino acid selection and activation, which are split into a large “core” domain (A_core_) and a much smaller “sub” domain (A_sub_), thiolation (T) domains as the amino acid carrier, condensation (C) domains for peptide bond formation, and sometimes additional modifying domains, such as epimerization (E) or methyltransferase (MT) domains (Süssmuth and Mainz 2017). Although individual NRPS systems make use of diverse strategies to broaden their product portfolio, for example, by using alternative starter modules (Rouhiainen et al. 2010), multispecific A domains (Meyer et al. 2016), or by skipping modules (Shishido et al. 2017), NRPSs are rather restricted in the generation of structural novelty. Although diversity of ribosomal peptides can be easily achieved by mutating codon triplets, the generation of diversity in NRPS systems requires a change in catalytic activity at the enzyme level, which is less likely to be attained during evolution—a restriction that, despite vigorous efforts, has severely hampered NRPS pathway engineering so far (Brown et al. 2018; Alanjary et al. 2019).

**Fig. 1.**
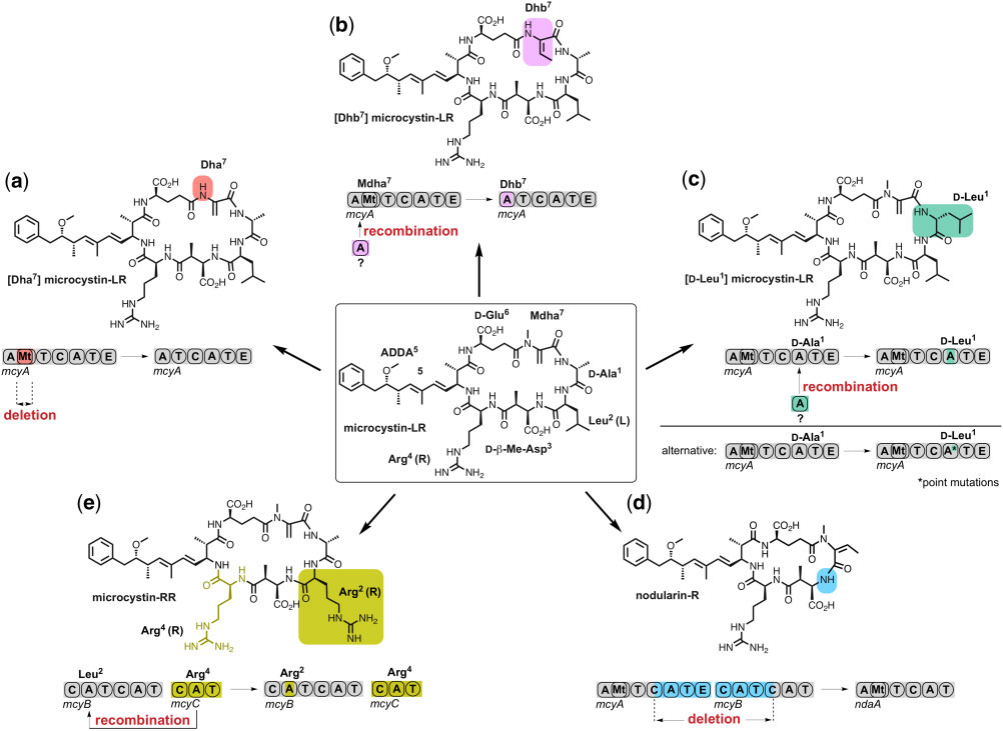
Evolution of microcystin diversity by recombination, DNA deletion, and point mutations. (*a*) Compared with microcystin-LR, [Dha^7^] microcystin-LR is produced by strains carrying a *mcyA* gene in which a segment encoding a *N*-methyltransferase got deleted (Fewer et al. 2008). (*b*) Recombination in the segment encoding the first A domain of *mcyA* likely gave rise to strains producing [Dhb^7^] microcystin-LR (Kurmayer et al. 2005). (*c*) Recombination in the segment encoding the second A domain of *mcyA* likely gave rise to strains producing [D-Leu^1^] microcystin-LR. Alternatively, point mutations have led to the same chemotype (Shishido et al. 2013). (*d*) Deletion of two modules encoded by *mcyA* and *mcyB* likely was involved in the evolution of microcystin-like nodularins (Rantala et al. 2004). (*e*) An intragenomic recombination event between *mcyB* and *mcyC* likely gave rise to the evolution of strains producing microcystin-RR (Fewer et al. 2007; Tooming-Klunderud et al. 2008; Meyer et al. 2016). Gene segments encoding modules are divided into adenylation (A), condensation (C), thiolation (T), and, if present, methylation (MT) domains. (M)dha, (*N*-methyl) dehydroalanine; Dhb, dehydrobutyrine.

This disparity raises the question of how these megasynthetases diverge in the course of evolution to facilitate the biosynthesis of the very diverse NRP cosmos. However, current basic models for the evolution of secondary metabolite gene clusters in general (Challis and Hopwood 2003; Fischbach et al. 2008) and NRPS in particular (Medema et al. 2014; Chevrette et al. 2020) are insufficient to explain in detail how the staggering diversity of NRPs has emerged. Although it is extensively noted that the modular nature of NRPS is predestined for diversification via recombination (Medema et al. 2014; Brown et al. 2018; Chevrette et al. 2020), all previous evolutionary studies are incidental. In consequence, a universal and systematic analysis is currently missing. Nevertheless, a growing number of reports hint to a lively mixing-and-matching of NRPS genes in the evolution of structural diversity (Fewer et al. 2007; Rounge et al. 2008; Ishida et al. 2009; Seyedsayamdost et al. 2012; Crüsemann et al. 2013; Shishido et al. 2013; Götze et al. 2019). Among these reports, by far the most intensively studied NRPS gene cluster with regard to evolution and diversification is the microcystin biosynthesis gene cluster (Meyer et al. 2016). Microcystins are widespread hepatotoxins with up to 100 known variants, which are produced by distantly related cyanobacterial genera (Dittmann et al. 2015). The cluster has not only diversified during the speciation process but also by a number of more recent inter- and intragenomic recombination events as well as point mutations and DNA deletions (Rantala et al. 2004; Kurmayer et al. 2005; Fewer et al. 2007, 2008; Tooming-Klunderud et al. 2008; Shishido et al. 2013), thereby making microcystin biosynthesis an excellent model system for the evolution of NRP diversity (fig. 1). In particular the sequence encoding the first A domain of *mcyB*, which is responsible for the incorporation of the amino acid at position 2, has been shown to be a recombination hotspot (Fewer et al. 2007; Meyer et al. 2016) (fig. 1*e*). Moreover, also positions 1 and 7 have been shown to have diversified by recombination of the underlying biosynthesis genes (Kurmayer et al. 2005; Shishido et al. 2013) (fig. 1*b* and *c*). The prevalence of recombination events in the evolution of microcystins raises the question whether the vast diversity observed in numerous NRP families has evolved similarly and whether defined evolutionary rules exist that could serve as blueprints for future NRPS engineering attempts.

By exploring a plethora of NRP structures and their producers’ genomes, we were able to trace back structural changes of dozens of compounds from multiple compound families to individual changes in NRPS genes. To assess recombination, we compared divergent biosynthetic genes by sliding window analysis to compute the average number of nucleotide differences per site between two sequences (π values). Segments with low π values (near 0) correlate with high homology of sequences, whereas segments with high π values (near 1) correlate with high divergence of sequences, which could be caused by recombination. Putative recombination events have further been validated by using Recombination Detection Program version 4 (RDP4) (Martin et al. 2015). We started our analysis at the phylum level, as cyanobacteria are an outstandingly valuable resource for studying natural recombination of NRPS genes (Welker and von Döhren 2006), due to extensive ecological monitoring on the metabolic and genomic level (Sogge et al. 2013; Agha and Quesada 2014; Mazur-Marzec et al. 2016) but later expanded our analysis to other phyla such as firmicutes and actinobacteria to exemplarily test whether the concept of recombination for NRP diversification is similarly widespread throughout the bacterial kingdom.

Our results show that recombination is a key driver in the evolution of bacterial NRPS across various phyla that directly translates into the structural diversity in the respective compound families. Moreover, they unveil an unprecedented, network-like mosaic structure of NRPS genes that goes beyond the boundaries of biosynthetic gene clusters and species, thereby providing crucial insights in bacterial ecology and evolution. Most surprisingly, recombination mainly targets A domains alone, causing partial substitutions in the A_core_ subdomain. These results allowed us to develop an universal evolutionary model for NRPS machineries that is in perfect agreement with recent structural insights in the catalytic cycle of NRPS (Süssmuth and Mainz 2017; Izoré and Cryle 2018) but strongly contradicts the widely believed hypothesis that A and C domains coevolve and are transferred together between modules (Lautru and Challis 2004; Baltz 2014). Furthermore, our results significantly challenge the attributed role of C domains as stringent selectivity filter during NRP synthesis—a presumption mainly deduced from in vitro studies that has persistently influenced NRPS engineering attempts of overall only very modest success over the last 20 years (Baltz 2014; Brown et al. 2018; Alanjary et al. 2019). Therefore, this first comprehensive survey of natural NRPS biocombinatorics is pivotal to our understanding of NRP biosynthesis from a mechanistic and evolutionary perspective and may guide future engineering approaches.

## Results

### Recombination Is Prevalent in NRPS Gene Clusters

**Fig. 2.**
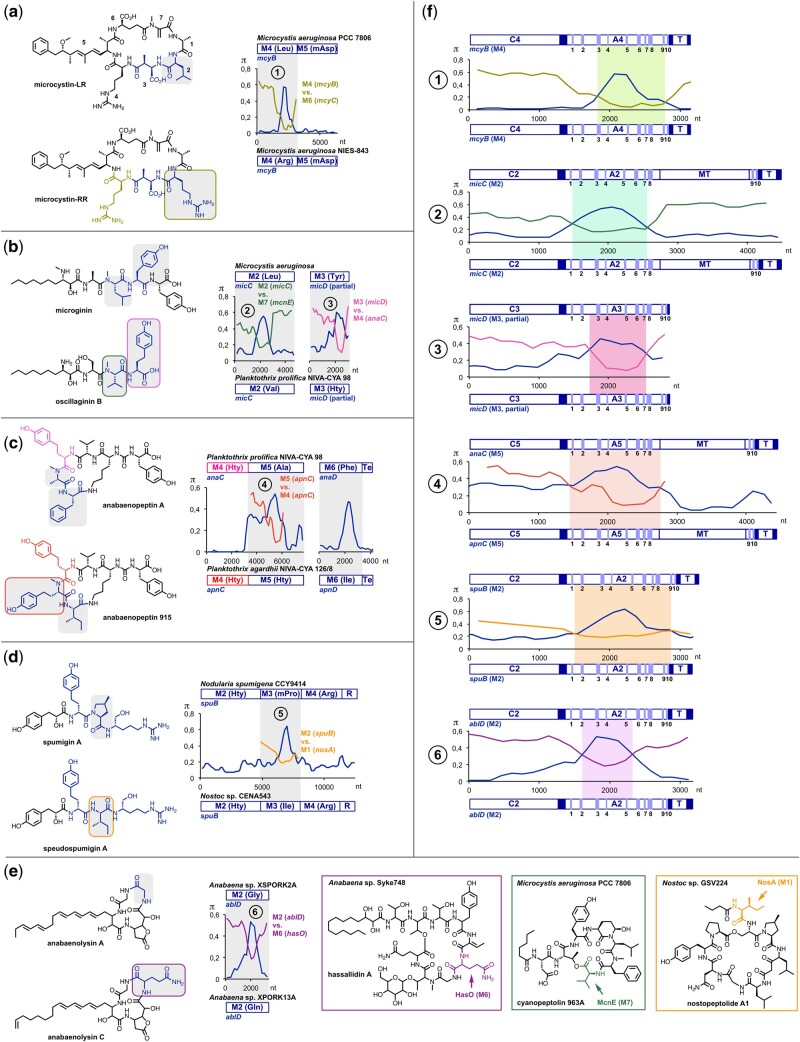
Diversification of cyanobacterial NRPs via recombination in the biosynthesis of (*a*) microcystins, (*b*) microginins, (*c*) anabaenopeptins, (*d*) spumigins, and (*e*) anabaenolysins. Structural differences between pairs from compound families (gray squares) correlate with nucleotide sequence divergence of the genes encoding NRPS modules (M). Related sequences have been aligned for pairwise comparison. π values (average number of nucleotide differences per site between two sequences) were computed using the sliding window mode in DnaSP (width, 300 nt; step, 150 nt). The mosaic structure of the genes (Smith 1992) clearly indicates recombination. This notion is also strongly supported by the detection of gene segments that complement divergent sites in a reciprocal fashion (numbered bullet points [BP] 1–6). Notably, the complement sequences stem from modules of the same cluster (BP 1, 4), from different clusters of the same species (BP 3), or from different clusters of different species (BP 2, 5, 6). Amino acid residues in the structures are color-coded to trace back their biosynthetic origin to individual modules. Hty, homotyrosine; Hph, homophenylalanine; mPro, 4-methylproline; mAsp, 3-methylaspartic acid; Te, thioesterase, R, reductive domain. (*f*) Close-up representation of putative recombination events to evaluate exchange unit boundaries. Gene segments encoding modules are divided into adenylation (A), condensation (C), thiolation (T), and, if present, methylation (MT) domains. Adenylation domain-specific core motifs are indicated by bands and numbers (1–10) (Marahiel et al. 1997). Linkers are indicated as filled squares. Highlighted parts of the graphs represent regions that are more closely related to sequences encoding other modules than to sequence of the respective ortholog.

In a previous study, we have biochemically dissected the impact of recombination events and point mutations on the diversification of microcystins (Meyer et al. 2016). The structural diversity of microcystins is dominated by a high variability of positions 2 and 4 (fig. 2*a*) (Welker and von Döhren 2006) and the gene encoding the A domain responsible for the incorporation of the variable amino acid at position 2 (McyB-A1) has been shown to be a recombination hotspot (Fewer et al. 2007). Most frequently, a stretch of sequence covering the region between the conserved motifs A3 to A9 (Marahiel et al. 1997) of the Arg-specific McyC module has been integrated into the nonsynonymous Leu-specific McyB module (fig. 2*a*) (Fewer et al. 2007; Meyer et al. 2016). This recurrent recombination event, together with relaxed substrate specificity of the resulting hybrid A domain, accounts for much of this compound family’s diversity. Remarkably, also positions 1 and 7 have been shown to have diversified by recombination of the underlying biosynthesis genes (supplementary fig. S1*a*, Supplementary Material online) (Kurmayer et al. 2005; Shishido et al. 2013), making recombination a major driver of microcystin diversification.

**Fig. 3.**
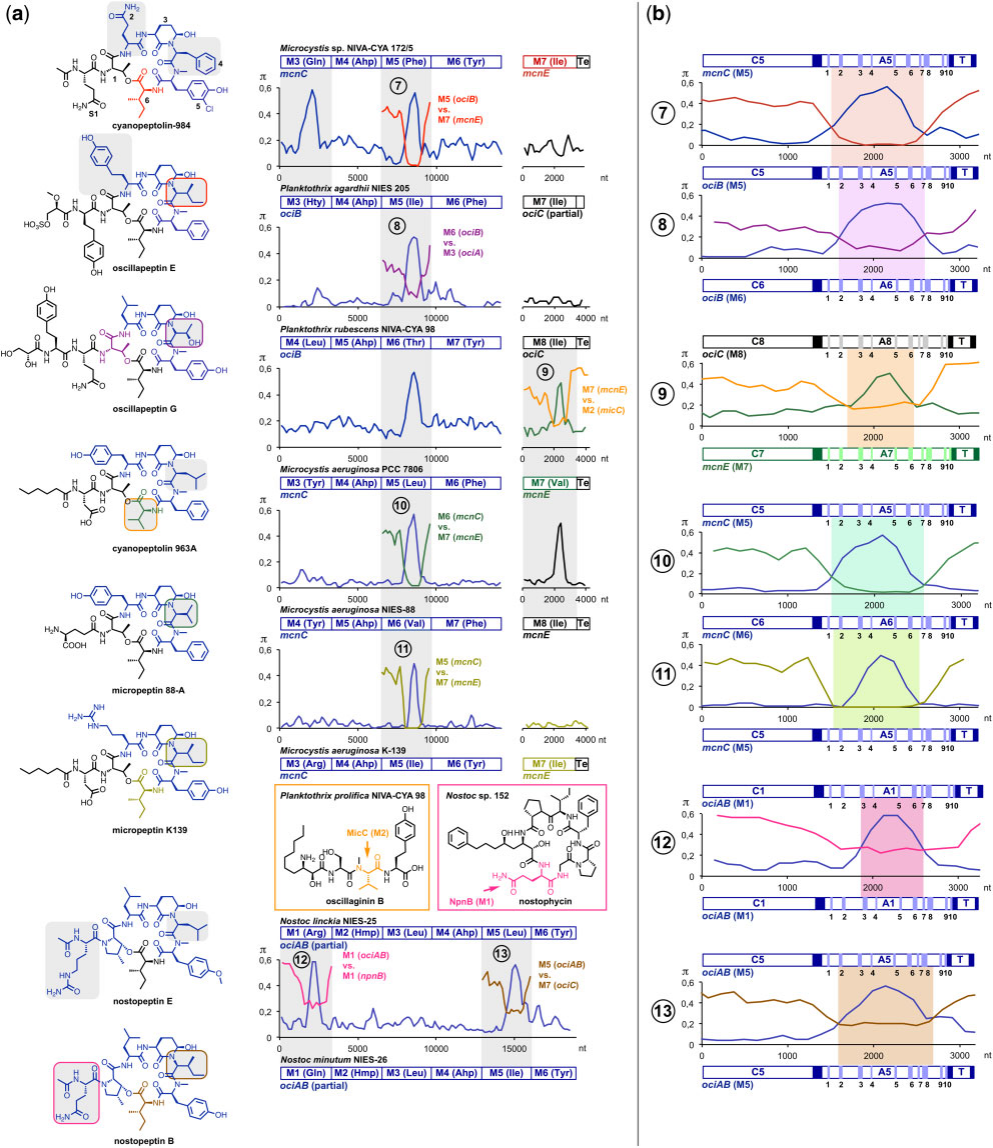
Diversification of Ahp-cyclodepsipeptides via recombination. (*a*) Structural differences of ahpcyclodepsipeptides (gray squares) correlate with nucleotide sequence divergence of the genes encoding NRPS modules (M). Closely related sequences have been aligned for pairwise comparison. π values (average number of nucleotide differences per site between two sequences) were computed using the sliding window mode in DnaSP (width, 300 nt; step, 150 nt). The mosaic structure of the genes (Smith 1992) clearly indicates recombination. This notion is also strongly supported by the detection of gene segments that complement divergent sites in a reciprocal fashion (BP 7–13). Notably, the complement sequences stem from modules of the same cluster (BP 8, 11, and 13), from related clusters of different species (BP 7 and 10) or from different clusters of different species (BP 9 and 12). Amino acid residues in the structures are color-coded to trace back their biosynthetic origin to individual modules. Ahp, 3-amino-6-hydroxy-2-piperidone; Hty, homotyrosine; Hmp, 3-hydroxy-4-methylproline; Te, thioesterase. (*b*) Close-up representation of putative recombination events to evaluate exchange unit boundaries. Gene segments encoding modules are divided into adenylation (A), condensation (C), and thiolation (T) domains. Adenylation domain-specific core motifs are indicated by bands and numbers (1–10) (Marahiel et al. 1997). Linkers are indicated as filled squares. Highlighted parts of the graphs represent regions that are more closely related to sequences encoding other modules than to sequence of the respective ortholog.

The prevalence of recombination in the evolution of microcystin diversity motivated us to investigate recombination events in bacterial NRPS genes systematically at the phylum level. Cyanobacteria are an outstandingly valuable resource for studying natural recombination of NRPS genes (Welker and von Döhren 2006), due to extensive ecological monitoring on the metabolic and genomic level (Sogge et al. 2013; Agha and Quesada 2014; Mazur-Marzec et al. 2016). Much interest on cyanobacterial metabolites stems not only from toxin-producing cyanobacterial blooms, which raise concerns of public health, but also from pronounced pharmacological potential of many compounds with diverse bioactivities. This leads to an increasing amount of data on closely related chemo-, eco-, and genotypes ready for comprehensive data mining. After analysis of diverse NRP families and the in-depth analysis of available genome sequences we were able to pinpoint 13 previously unrecognized recombination events, together with four previously reported events (Ishida et al. 2009; Christiansen et al. 2011), by correlating structural differences between pairs from compound families with nucleotide sequence divergence of the genes encoding NRPS modules. Moreover, in many cases we detected gene segments that complement these divergent sites, thereby revealing a mosaic structure of the genes (Smith 1992), a clear indication of recombination. These putative recombination events led to changes in the amino acid composition of microginins, anabaenopeptins, spumigins, anabaenolysins, Ahp-cyclodepsipeptides, and aeruginosins (figs. 2 and 3; supplementary fig. S1*c*, Supplementary Material online). Intriguingly, for 12 of these events, we were able to identify plausible recombination partner sequences from characterized NRP biosynthesis genes, which either stem from modules of the same cluster (fig. 2, bullet point [BP]4; fig. 3, BP8, 11, and 13), from related clusters of different species (fig. 3, BP7 and 10), from different clusters of the same species (fig. 2, BP3), or from different clusters of different species (fig. 2, BP2, 5, and 6; fig. 3, BP9 and 12). To get further support for recombination, we used RDP4 (Martin et al. 2015). By using multiple recombination detection methods (RDP [Martin and Rybicki 2000], GENECONV [Padidam et al. 1999], Bootscan [Salminen et al. 1995], Maxchi [Smith 1992], Chimaera [Posada and Crandall 2001], SiSscan [Gibbs et al. 2000], 3Seq [Boni et al. 2007], LARD [Holmes et al. 1999]) we obtained strong support for recombination in all events for which we could comprehensively identify plausible recombination partner sequences, because recombination could be detected in all cases with all methods used (supplementary figs. S2–S13, Supplementary Material online). It is known that different methods assessing recombination lead to different results depending on factors such as sequence divergence. Therefore, different methods should be used to attain maximum power while minimizing false positive results (Posada and Crandall 2001).

With eight documented cases of recombination, the family of Ahp-cyclodepsipeptides stands out in our data set (fig. 3). This compound family with currently more than 200 members, all of which possess an unique 3-amino-6-hydroxy-2-piperidone (Ahp)-moiety at position 3 is exceptionally diverse (Köcher et al. 2020). Besides the Ahp-moiety, these remarkably active serine protease inhibitors share a very conserved ring topology in which highly conserved positions (1, 3, 5) alternate with highly (2, 4) or at least slightly (6) flexible ones (fig. 3*a*) (Welker and von Döhren 2006). Our data show that recombination contributes to diversification of all flexible positions (fig. 3). However, the results also clearly indicate that the module responsible for incorporation of the amino acid at position 4 is a recombination hotspot, whereas the most variable position of Ahp-cyclodepsipeptides, position 2 (Welker and von Döhren 2006), seems to be much less frequently altered by recombination (fig. 3).

**Fig. 4.**
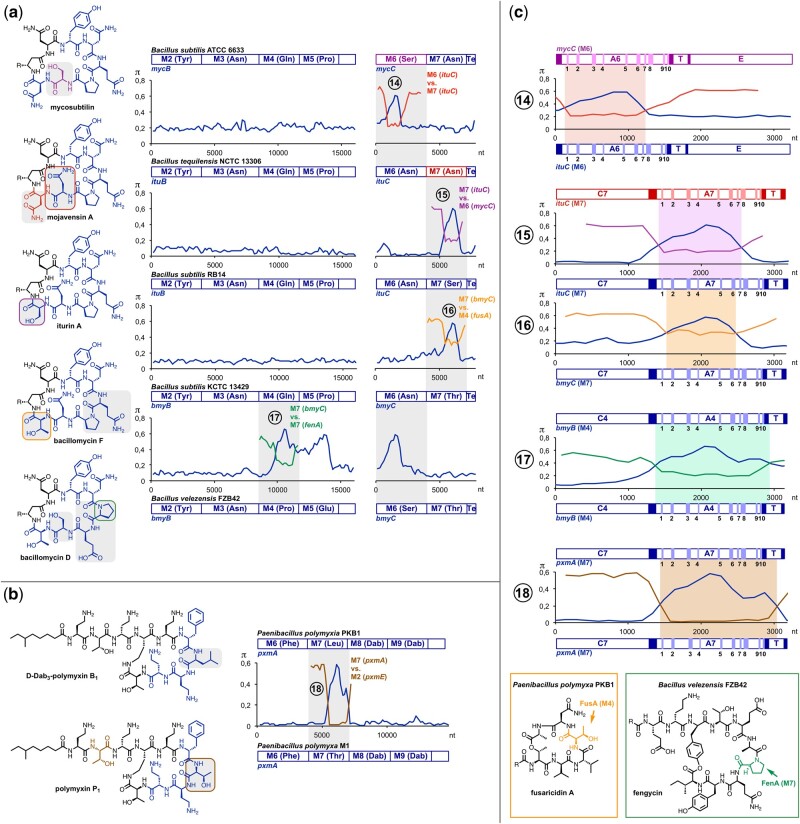
Diversification of noncyanobacterial NRPs via recombination. Putative recombination events in the biosynthesis of (*a*) iturinic lipopeptides and (*b*) polymyxins. Structural differences of NRPs (gray squares) correlate with nucleotide sequence divergence of the genes encoding NRPS modules (M). Closely related sequences have been aligned for pairwise comparison. π values (average number of nucleotide differences per site between two sequences) were computed using the sliding window mode in DnaSP (width, 300 nt; step, 150 nt). The mosaic structure of the genes (Smith 1992) clearly indicates recombination. This notion is also strongly supported by the detection of gene segments that complement divergent sites in a reciprocal fashion (numbered bullet points [BP] 14–18). Notably, the complement sequences stem from modules of the same cluster (BP 14 and 18), from related clusters of different species (BP 15), from different clusters of the same species (BP 17), or from different clusters of different species (BP 16). Amino acid residues in the structures are color-coded to trace back their biosynthetic origin to individual modules. Dab, diaminobutyric acid; Te, thioesterase; R, alkyl moiety. (*c*) Close-up representation of putative recombination events to evaluate exchange unit boundaries. Gene segments encoding modules are divided into adenylation (A), condensation (C), and thiolation (T) domains. Adenylation domain-specific core motifs are indicated by bands and numbers (1–10) (Marahiel et al. 1997). Linkers are indicated as filled squares. Highlighted parts of the graphs represent regions that are more closely related to sequences encoding other modules than to sequence of the respective ortholog.

Next, we turned our attention to prolific NRP producers from other phyla such as firmicutes and actinobacteria to exemplarily test whether the concept of recombination for NRP diversification is similarly widespread throughout the bacterial kingdom. In both phyla together we were able to detect 11 previously unrecognized recombination events in the biosynthesis of iturinic lipopeptides, polymyxins, and glycopeptide antibiotics, together with a previously reported event from hormaomycin biosynthesis (Crüsemann et al. 2013) (fig. 4 and supplementary fig. S1, Supplementary Material online). For 5 of these 12 events we were able to identify plausible recombination partner sequences from characterized NRP biosynthesis genes, which either stem from modules of the same cluster (fig. 4, BP14 and 18), from related clusters of different species (fig. 4, BP15), from different clusters of the same species (fig. 4, BP17), or from different clusters of different species (fig. 4, BP16). Again, analysis with RDP4 gave strong support for recombination in all events for which we could comprehensively identify plausible recombination partner sequences, as recombination could be detected in all cases with all methods used (supplementary figs. S14–S17, Supplementary Material online).

Together, these results show that recombination is a key driver in the evolution of NRP diversity that is very widespread in the bacterial kingdom. The number of detected recombination events in an individual compound family roughly correlates with the number of known compounds and sequenced biosynthesis gene clusters for all phyla investigated, thereby indicating that recombination is an abundant and ubiquitously occurring phenomenon in the biosynthesis of NRPs.

### The A_core_ Domain Is a Diversification Hotspot

To test whether the widespread occurrence of recombination follows defined evolutionary rules, we analyzed exchange unit boundaries of individual recombination events on the DNA level (figs. 2*f*, 3*b*, and 4*c*) as well as on the protein level (supplementary figs. S18 and S19, Supplementary Material online). Therefore, a sliding window analysis was used to identify breakpoints that mark closer relationships to sequences encoding other modules than to sequence of the respective ortholog. Very remarkably, recombination targets predominantly the A_core_ domain to achieve the exchange of individual amino acids in NPR scaffolds. The only exceptions could be found in the biosynthesis of an anabaenopeptin (fig. 2, BP4) and an iturinic lipopeptide (fig. 4, BP17), for which in the first case a C–A didomain and in the second case an A–T–C–A multidomain seems to be exchanged. Intriguingly, also in these cases, A subdomain swaps seem to contribute to compound diversification. This stunning observation points to more complex recombination scenarios in which multiple recombination events contributed to the diversification of NRPS genes. However, the more or less exclusive evolutionary focus on the A_core_ domain strongly contradicts the widely believed hypothesis that A and C domains coevolve and are transferred together between modules (Lautru and Challis 2004; Baltz 2014).

**Fig. 5.**
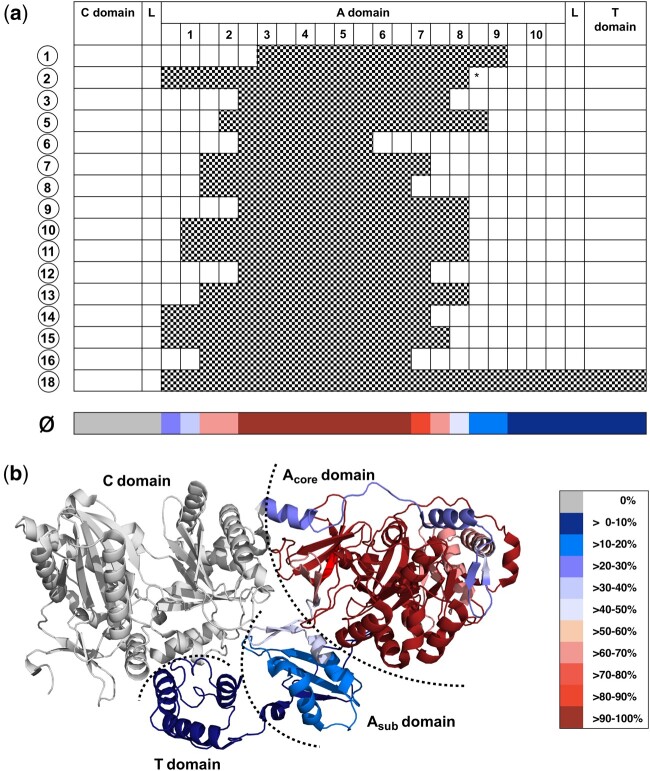
Visualization of exchange unit boundaries in NRPS modules. (*a*) Schematic visualization of the deduced exchange units (supplementary figs. S18 and S19, Supplementary Material online) that most likely result from a single recombination event (checked pattern). Modules are divided into adenylation (A), condensation (C), thiolation (T) domains, and linkers (L). Adenylation domain-specific core motifs are indicated by numbers 1–10 (Marahiel et al. 1997). Modules that possess an additional methyltransferase (MT) domain between core motif 8 and 9 are marked with an asterisk. The plurality of exchange unit boundaries indicates a pronounced plasticity of the Acore domain, which provides multiple breakpoints for subdomain swaps to be harnessed by evolution. (*b*) Projection of the deduced exchange units on the structure of SrfA–C (Tanovic et al. 2008) illustrates the obvious trend to keep the native C–A linker, the Asub domain and consequently the Asub–T domain interface intact.

Projection of the deduced exchange units (fig. 5*a*) on the structure of SrfA-C (Tanovic et al. 2008) illustrates the very obvious trend to keep the native C–A linker, the A_sub_ domain and consequently the A_sub_–T domain interface intact (fig. 5*b*). However, within these limitations exchange unit boundaries are remarkably diverse. This plurality indicates a pronounced plasticity of the A_core_ domain, which provides multiple breakpoints for subdomain swaps to be harnessed by evolution (fig. 5*a*).

Intriguingly, A subdomain exchanges seem to follow a quite complementary scheme compared with recombination events that lead to the integration of E domains in NRP pathways, which change the configuration of the amino acid that is incorporated by the module from l- to d-configuration (Rounge et al. 2008). In these events, special T and C domains (T_E_ and ^D^C_L_) replace the conventional T and C domains (T_C_ and ^L^C_L_) leading to the exchange of T_C_–^L^C_L_ didomains with T_E_–E–^D^C_L_ tridomains (supplementary fig. S20, Supplementary Material online). Notably, the A_sub_ domain of the adjacent A domain gets exchanged, too, thereby also indicating the importance of native A_sub_–T domain interfaces in functional NRPS architectures.

## Discussion

Current understanding of the diversification of NRP pathways is largely based on the chemical structures of bioactive compounds (Welker and von Döhren 2006), whereas the evolutionary mechanisms driving their remarkable chemical diversity are poorly understood (Calteau et al. 2014). Previous studies have mainly focused on single pathways or do not link genotype with chemotype data. This starts to change with the growing number of accessible genomes that can be compared to unravel the evolutionary history of compound families, together with community efforts to collect and catalog data on biosynthesis gene clusters (Kautsar et al. 2020), chemical structures of natural products (van Santen et al. 2019), or genotype/chemotype links (Schorn et al. 2020). Here, we present substantial evidence that A_core_ subdomain swapping via recombination is a very widespread phenomenon that considerably contributes to the diversification und functionalization of NRP families.

One impressive example of the subtle interplay between diversification, functionalization, and natural selection can be seen in the family of antiproteolytic Ahp-cyclodepsipeptides, for which our data indicate a huge discrepancy in the diversification of positions 2 and 4 via recombination. This finding is remarkable in light of Ahp-cyclodepsipeptide’s mode of action. Several structural and biochemical studies have shown that the amino acid side chain of position 2 occupies the S1 site, which mainly determines the specificity of the respective protease subtype like chymotrypsin, trypsin, or elastase (Köcher et al. 2020), whereas the amino acid side chain of position 4 occupies the S2’ site and modulates the potency and selectivity of serine protease inhibitors for certain isoforms (de Veer et al. 2015). Therefore, the obvious bias in the diversification of particular amino acid positions in the Ahp-cyclodepsipeptide scaffold, either by point mutations like predominantly in case of position 2 or by recombination like in case of position 4, suggests different evolutionary strategies to fine-tune NRP bioactivities: Point mutations in general are leading to much more conservative changes but have the benefit to frequently maintain basic activity while screening for advantageous mutations (e.g., via A domains that gain multispecificity due to a mutation that leads to a more relaxed substrate specificity). In contrast, recombinations can efficiently lead to more substantial changes, thereby creating evolutionary shortcuts in modulating existing bioactivities. The first assumption is supported by the fact that many strains produce various Ahp-cyclodepsipeptides with several different amino acids at position 2, which indicate a widespread occurrence of multispecific A domains in the biosynthesis of this position (Köcher et al. 2020). Moreover, it seems that recombination events that lead to the incorporation of Ile at position 4 happened several times in the course of evolution (fig. 3, BP7, 11, and 13). This remarkable case of convergent evolution points to the specific importance of this amino acid in the Ahp-cyclodepsipeptide scaffold. This notion is strongly supported by a recent extensive screening of the S2’ site specificity of 13 different serine proteases with a synthetic inhibitor library that revealed an overall preference for Ile, which is also present at the complementary site in many naturally occurring serine protease inhibitors (de Veer et al. 2015). Therefore, although the activity of Ahp-cyclodepsipeptides on individual subtype isoforms has not been investigated so far, their evolution seems to mirror large-scale artificial screening campaigns.

**Fig. 6.**
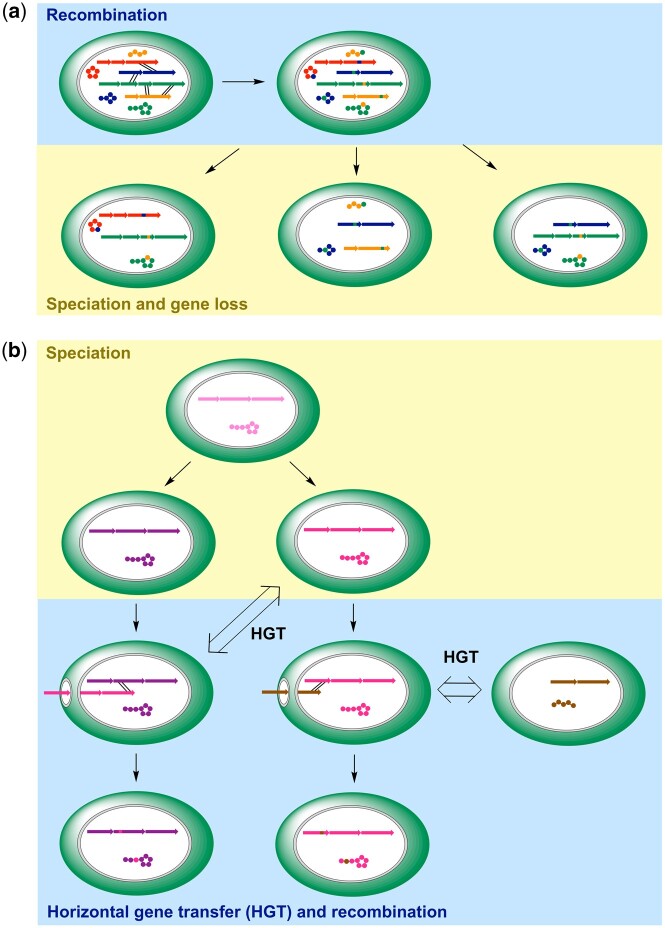
Unifying model for the evolution of the present-day variety of NRPs (simplified with amino acids as beads on a string) using the example of cyanobacteria. (*a*) Intragenomic recombination in last common ancestors that harbored a variety of NRPS gene clusters led to the diversification of multiple compound families. After speciation, many clusters have been lost in individual species due to genome streamlining. This could explain the patchy distribution of NRPS families as well as the unprecedented, network-like mosaic structure of NRPS genes, which is exemplified by the high proportion of putative recombination pairs from different clusters of different species or even different genera (fig. 2, BP2, 5, and 6; fig. 3, BP9 and 12). Similarly, intragenomic recombination in present-day species continuously contributes to generation of structural variants (e.g., fig. 2, BP1 and 4; fig. 3, BP8, 11, and 13). (*b*) Additionally, horizontal gene transfer (HGT) together with recombination likely drives the diversification of NRPS families, either between related clusters of different species (e.g., fig. 3, BP7 and 10) or between different clusters of different species.

From an evolutionary point of view, the most striking observation is the high proportion of putative recombination pairs from different clusters of different species or even different genera (fig. 2, BP 2, 5, and 6; fig. 3, BP 9 and 12; fig. 4, BP 16) leading to an unprecedented, network-like mosaic structure of NRPS genes. Two general hypotheses could explain this phenomenon. First, horizontal gene transfer followed by recombination could have led to the mosaic pattern. Second, a last common ancestor could have harbored a variety of NRPS gene clusters ready to recombine, which later have been partly lost in the course of speciation (fig. 6). A strong argument for the second hypothesis is that, although a direct relationship of the exchanged sequences in the intercluster/interspecies events is obvious, the associated π values (average number of nucleotide differences per site between two sequences) are relatively high in comparison to other recombination events (figs. 2–4). These results indicate that the respective sequences might not represent recent donor/recipient pairs but descendants from more ancient recombination events. This, together with the fact that frequency of homologous recombination decreases sharply with declining taxonomic relatedness between donor and recipient (Majewski and Cohan 1999), strongly advocates the theory of an ancient superproducer. In line with this is the finding that repeated loss of individual gene clusters rather than horizontal gene transfer is responsible for the sporadic distribution of microcystin (Rantala et al. 2004) and very likely also a number of other NRP families like aeruginosins (Ishida et al. 2009) or Ahp-cyclodepsipeptides (Rounge et al. 2007) among modern cyanobacteria. The genus *Microcystis*, for example, is known to produce microcystins, microginins, anabaenopeptins, Ahp-cyclodepsipeptides, and aeruginosins, whereas production of peptides from these five classes in individual strains of *Microcystis* range from four to none (Welker and von Döhren 2006). As it can be assumed that the chemotype for these five classes directly reflects the genotype (Welker and von Döhren 2006), genome streamlining seems to be a widespread phenomenon. On the other hand, we also see occasional signs for the first hypothesis as in the biosynthesis gene cluster of oscillapeptin E. Here, parts of module 5 have a much more pronounced sequence similarity to module 7 of a distant relative than to the intracluster counterpart (fig. 2, BP1; supplementary fig. S21, Supplementary Material online). This clearly indicates a recombination event with horizontally acquired genes. In summary, the current variety of compounds and the mosaic-like pattern observed in biosynthesis genes as well as in NRPS family distribution likely reflect the ongoing evolution of NRPs as gene collectives in a transforming genetic background shaped by genome streamlining as well as horizontal gene transfer (fig. 6).

However, it should be taken into account that our perception of recombination events is biased as the available genome data represent just a small fraction of the microbial biodiversity and the direction of the recombination events in closely related strains often is subject to interpretation. Therefore, our deduced evolutionary snapshots inherently cannot reflect reality in all cases. However, they do provide plausible trajectories that allow for the first time to build a unifying model for NRPS evolution (fig. 6) that will continue to refine with the growing number of published genome sequences and discovered NRP congeners.

From a structural and mechanistic perspective, it is intriguing that predominantly only the A_core_ domain is targeted to achieve the exchange of individual amino acids in NPR scaffolds. Exchange unit boundaries within in the A_core_ domain are remarkably diverse (fig. 5), which might be a consequence of the large number of highly conserved core regions (A1–A10) serving as putative recombination hotspots. However, there is a very obvious trend to keep the native C–A linker, the A_sub_ domain and consequently the A_sub_–T domain interface intact (fig. 5). Crystal structures of multimodular NRPS suggest that the linker between C and A domains is critical for forming a productive interface during the catalytic cycle by contributing to the formation of a stable catalytic platform (Brown et al. 2018). Therefore, selection of recombination events that maintain the structural integrity of the C–A interface appears plausible, although recent engineering studies have proofed that the C–A linker can be exploited for the reengineering of domains (Bozhüyük et al. 2018; Calcott et al. 2020; Kaniusaite et al. 2020). On the other hand, homologous recombination has a strong inherent bias to favor sequences with high sequence similarity (Majewski and Cohan 1999). This might also explain the preference to recombine in the proximity of highly conserved domain-specific core motifs (e.g., A1–A8), which in turn excludes the flexible C–A domain linker in recombination events that target the substrate binding pocket of A domains but includes the linker when exchanging T_C_–^L^C_L_ didomains with T_E_–E–^D^C_L_ tridomains (A8 of the first module to A1 of the adjacent module; supplementary fig. S20*a*, Supplementary Material online). However, this bias cannot explain the tendency to leave the native A_sub_–T domain interface intact in both scenarios, because the highly conserved A10 motif (NGK(V/L)DR) together with the neighboring conserved LPxP motif (Miller et al. 2014) (sometimes regarded as A11) (Süssmuth and Mainz 2017) marks the junction to the A–T domain linker and therefore the encoding DNA sequence would make a perfect recombination point. Thus, our results imply that the A_sub_–T domain interface is very critical for correct function and that strong selection is present to preserve it. The first deduction is heavily supported by recent crystal structures of A domains in different catalytic states, which show that although the A_core_ domain is relatively well constrained, the C_sub_ domain rotates substantially relative to A_core_ in the catalytic cycle. This so-called domain alternation reorganizes the A_core_–A_sub_ interface via the well-conserved A8 hinge motif. The resulting rigid-body torsion of the A_sub_ domain of approximately 140° also relocates the aminoacylated holo-T domain, thereby allowing the substrate to traverse long distances between domain active sites (Süssmuth and Mainz 2017; Izoré and Cryle 2018). This led to the assumption that A_sub_ is the centerpiece of NRPS machineries (Süssmuth and Mainz 2017). Further experimental support for the importance of A_sub_–T domain interfaces in controlling domain conformations comes from biochemical studies on EntF in which mutations in the conserved LPxP motif at the N-terminus of the A–T domain linker region led to severely impaired production of enterobactin (Miller et al. 2014). This motif forms hydrophobic interactions with the A_sub_ domain and is therefore of structural importance for the A domain as well as for the affiliated T domain (Miller et al. 2014; Süssmuth and Mainz 2017). Finally, there are plenty of interrupted A domains that harbor auxiliary domains such as methyltransferases, ketoreductases, oxidases, and monooxygenases, which are most commonly inserted between core motifs A8 and A9 (fig. 2*e*, BP2 and 4), but also A2 and A3, or A4 and A5 (Labby et al. 2015). These enzyme-in-an-enzyme architectures, which on the first glance are so odd that they were initially believed to be inactive (Labby et al. 2015), might be the living proof for nature’s effort to keep native C–A, A_sub_–T, and T–C domain interfaces intact while implementing novel enzyme functionalities.

Notably, there was an exception in our data set for which the A_sub_ domain was exchanged together with a large part of the A_core_ domain (supplementary fig. S19, BP 18, Supplementary Material online). However, in this case part of the T domain was exchanged as well, thereby preserving the A_sub_–T domain interface of the donor system.

The observed predominance of A_core_ subdomain swaps in the diversification of NRP biosynthesis pathways strongly contradicts the widely believed hypothesis that A and C domains coevolve and are transferred together between modules (Lautru and Challis 2004; Baltz 2014). Moreover, they vigorously challenge the attributed role of C domains as stringent selectivity filter during NRP synthesis. This hypothesis was first deduced from in vitro studies that bypassed the editing function of adenylation domains (Belshaw et al. 1999) and later was fueled by an increasing amount of unsuccessful engineering attempts (Brown et al. 2018). Moreover, in glycopeptide biosynthesis it has been shown that the modification of amino acids after the activation of the A domain by *trans*-acting enzymes is controlled by the selectivity of the upstream condensation domain (Kaniusaite et al. 2019). However, a general strict selectivity would contradict the multitude of productive recombination events without the concomitant exchange of C domains (figs. 2–4 and supplementary fig. S1, Supplementary Material online), as fortuitous multispecificity in all presented cases seems highly unlikely. This is supported by the finding that in recombination events that integrate an E domain into a pathway exchange of the adjacent ^L^C_L_ domain with a ^D^C_L_ domain seems mandatory (supplementary fig. S20, Supplementary Material online), thus supporting the presumed role of C domains as stereochemical gatekeepers (Süssmuth and Mainz 2017). The idea that C domains have a pronounced role as stereochemical gatekeepers but not as selectivity filters is supported by extensive C domain phylogenies in which related C domains do cluster according to the stereochemistry of their substrates (^L^C_L_ vs. ^D^C_L_) but, in strong contrast to their A domain counterparts, do not cluster according to their assumed substrate specificity (Rausch et al. 2007). All together, these novel insights give rise to serious doubts whether a “specificity conferring code equivalent to that of A domains” (Süssmuth and Mainz 2017) exists. Further support against the “prevailing dogma” of C domain substrate specificity comes from a very recent study centered on A domain reengineering, in which the authors could generate novel NRPs by substitution of A domains alone (Calcott et al. 2020). However, because of the overall very high sequence similarity of homologous C domains adjacent to the diverged A domains our data would provide a rich source to search for putative specificity-shifting mutations.

Notably, an exception to the predominant A_core_ subdomain exchanges has recently been reported for the biosynthetic pathway of virginiafactin A–D from *Pseudomonas* sp. QS1027 (Götze et al. 2019). There a C–A didomain (or even a T–C–A–T multidomain) exchange gave rise to diversification in the syringafactin lipopeptide family, once again showcasing that nature’s evolutionary trajectories may indeed be very multifaceted. However, the observation that in the majority of cases domains that account for the structural diversity of a product are subject to recombination is very similar to what has been reported for type I polyketide synthases (PKS), another major group of modular megasynthases (Jenke-Kodama et al. 2005, 2006; Jenke-Kodama and Dittmann 2009). Dissection of nucleotide sequences encoding modules of various PKS clusters from *Streptomyces avermitilis*, for example, revealed incongruities in the phylogenetic pattern of their individual acyltransferase (AT), ketoreductase (KR), dehydratase (DH), and enoylreductase (ER) domains, which are responsible for substrate selection and the degree of reduction of the carbon chain. In contrast to that, incongruities have not been observed for ketosynthase (KS) domain sequences, which encode the domains responsible for condensation reactions in polyketide biosynthesis. Phylogenetic trees further suggested that these incongruities result from recombinational replacements within and between biosynthetic gene clusters of *S. avermitilis* and putative sites for homologous recombination were discovered in the interdomain regions of PKS modules as well as within domains (Jenke-Kodama et al. 2005, 2006). Moreover, for *trans*-AT PKS—PKS that lack integrated AT domains—gene clusters appear to be patchworks acquired from diverse sources and assembled by multiple recombinatorial events (Nguyen et al. 2008). Therefore, it seems that the evolution of multimodular assembly lines as different as NRPS and PKS share many common traits.

Besides providing significant conceptual advances in our understanding of NRPS evolution and presenting profound new molecular-level insights into the mechanisms or NRP diversification, our study is also of utmost relevance for NRPS engineering by means of synthetic biology. Besides an overall rather disappointing success rate of NRPS engineering approaches (Brown et al. 2018; Alanjary et al. 2019), there have been very successful attempts that focused on highly conserved motifs like the active site motif of C domains (HHXXXDG) (Yakimov et al. 2000), or rigid linkers like the subdomain linker of C domains to manipulate NRPS on the module level (Bozhüyük et al. 2019). Although one explanation for the high success rates of these approaches could undeniably be the modulation of putative C-domain specificities, especially on the acceptor site, a more simple explanation could be minimized interfering in major domain–domain interactions during the NRPS catalytic cycle by keeping highly dynamic linker regions and interfaces intact (Bozhüyük et al. 2019). With regard to the latter the observed subdomain swapping in the evolution of NRP pathways could be seen as a much more parsimonious version of this strategy, which holds great potential for future engineering approaches. The minimally invasive concept of subdomain swaps is in stark contrast to a plethora of NRPS engineering attempts that focused on the exchange of whole domains, multiple domains or entire modules and despite great efforts led to disappointingly few success stories (Brown et al. 2018; Alanjary et al. 2019). Most of these trial and error attempts ignored evolutionary schemes despite the current recognition that evolutionary informed pathway engineering is key to the artificial expansion of the natural product cosmos (Fisch et al. 2011; Sugimoto et al. 2014; Wlodek et al. 2017; Awakawa et al. 2018; Peng et al. 2019). The natural abundance of NRP congeners demonstrates that evolution has solved the problem of how to effectively recombine NRPS genes on innumerable occasions (Ackerley 2016). While NRPS engineering in the early days suffered from a very limited availability of sequence data, the exponentially growing compilation of sequences in the postgenomic era provides plenty of evolutionary snapshots for inspiration. Three pioneering studies that experimentally explored the potential of subdomain swapping in A domain engineering already indicated the huge potential of this concept (Crüsemann et al. 2013; Kries et al. 2015; Meyer et al. 2016). One study focused more on structural aspects and identified a flavodoxin-like subdomain responsible for substrate binding(Kries et al. 2015); the other studies, on the other hand, were inspired by putative recombination points in the hormaomycin pathway (supplementary fig. S1*f*, Supplementary Material online) (Crüsemann et al. 2013) as well as the microcystin pathway (fig. 2*a*) (Meyer et al. 2016). However, although very successful in emulating putative natural recombination events (Crüsemann et al. 2013), expansion of the concept to artificial combinations failed for most of the investigated domain swaps (Crüsemann et al. 2013; Kries et al. 2015). Comparison of the exchange unit boundaries of both studies that aimed for broader application with the extensive set of recombination events presented here revealed, that both approaches were very conservative, minimizing the exchange solely on the substrate binding pocket (Crüsemann et al. 2013; Kries et al. 2015). In contrast to that, it seems that a much longer part of the A_core_ domain is exchanged in most of the cases of natural recombination (supplementary fig. S22, Supplementary Material online). A likely reason for this could be that as long as the C–A domain junction is intact and the dynamic A_sub_–T domain core is unaffected a near full A_core_ substitution might influence the overall topology less than a mixed A_core_ domain. This might be even more pronounced if similarity of donor and acceptor A domain decreases, which was by far the most limiting factor in both studies (Crüsemann et al. 2013; Kries et al. 2015). Therefore, our study provides detailed insights in plenty of field-tested subdomain exchanges, which may guide future NRPS engineering approaches.

## Materials and Methods

### Analysis of Putative Recombination Events

To trace back putative recombination events, all characterized cyanobacterial biosynthetic gene clusters and their corresponding products were systematically screened for structural differences within NRP compound families as well as sequence divergence in homologous gene clusters. Additionally, structural variants that have not been assigned to biosynthetic gene clusters were detected by manually screening databases like NP atlas (van Santen et al. 2019) and Dictionary of Natural Products as well as an extensive set of literature. For orphan compounds, NCBI GenBank was checked for genomic information of the producers in question. If genomic information was present putative biosynthesis gene clusters were inferred by using antiSMASH (Blin et al. 2019) (for details on compounds, genes, and proteins analyzed in this study, see supplementary table S1, Supplementary Material online). Next, structural differences between compound pairs were correlated with nucleotide sequence divergence of the genes encoding NRPS modules. Therefore, π values (average number of nucleotide differences per site between two sequences) were computed in the sliding window mode in DnaSP 6 (Rozas et al. 2017) for sequence pairs that had been prealigned with Mega X (Kumar et al. 2018). The sliding window had a width of 300 nt and a step size of 150 nt. Plausible recombination partner sequences from characterized NRP biosynthesis genes have been identified with BlastN (Camacho et al. 2009). For visualization of sequence similarity/divergence and the analysis of putative recombination breakpoints, π values were plotted against window midpoints with Microsoft Excel. Putative recombination events have further been validated with the help of RDP4 (Martin et al. 2015), by using multiple recombination detection methods (RDP [Martin and Rybicki 2000], GENECONV [Padidam et al. 1999], Bootscan [Salminen et al. 1995], Maxchi [Smith 1992], Chimaera [Posada and Crandall 2001], SiSscan [Gibbs et al. 2000], 3Seq [Boni et al. 2007], LARD [Holmes et al. 1999]). Genes have been drawn to scale by using Illustrator for Biological Sequences (IBS) (Liu et al. 2015). The same systematic approach was then expanded to prolific NRP producers from other phyla such as firmicutes and actinobacteria for which representative NRP compound families together with their corresponding biosynthesis gene clusters have been analyzed accordingly. For details on compounds and biosynthesis genes that have been analyzed in this study, see supplementary table S1, Supplementary Material online.

### Analysis of Exchange Unit Boundaries

Module boundaries have been inferred by using the PKS/NRPS Analysis Website of the University of Maryland (Bachmann and Ravel 2009). Boundaries of domains, linkers, and core motifs have been inferred manually by sequence comparison to SrfA-C (Tanovic et al. 2008). For comparison of A domain pairs, sliding window analysis was executed on the protein sequences. The sequences were first aligned using MUSCLE (Edgar 2004). The gaps identified by the alignment were replaced with “X.” The resulting sequences were then separated into individual files using faSplit utility (http://hgdownload.cse.ucsc.edu/admin/exe/linux.x86_64/, last accessed February 2, 2021) and then fragmented using pyfasta tool (https://pypi.org/project/pyfasta/, last accessed February 2, 2021) ensuring with a sliding window of 50aa (-k flag) with 25aa overlap (-o flag) between windows. The resulting windows for each protein sequence were then subjected to BlastP (Camacho et al. 2009). The tabulated results were then filtered to retain matches by fragment number sequentially. BlastP output tables were manipulated in R 3.6.3(2013) using dplyr package (v0.8.5) (Wickham et al. 2018). The window midpoint versus percent ID plots were generated with Microsoft Excel to visualize the breakpoints of diversification and recombination. A reproducible workflow to implement this analysis is available at https://github.com/somakchowdhury/mwa-secmet-bgc (last accessed February 2, 2021) with its respective documentation at https://mwa-secmet-bgc.readthedocs.io/en/latest/index.html (last accessed February 2, 2021). Proteins have been drawn to scale by using IBS (Liu et al. 2015). Structural models were predicted by the Phyre2 web portal for protein modeling, prediction, and analysis (Kelley et al. 2015). For details on enzymes that have been analyzed in this study, see supplementary table S1, Supplementary Material online.

### Code Availability

All code and software used in this study are described and/or are available in the Materials and Methods section.

## Supplementary Material

Supplementary data are available at *Molecular Biology and Evolution* online.

## Supplementary Material

msab015_Supplementary_Data
