# Supplementary material for: The Landscape of Recombination Events That Create Nonribosomal Peptide Diversity
Source: Mol Biol Evol. 2021 Jan 22;38(5):2116–30. doi: 10.1093/molbev/msab015 (PMC8097286; doi:10.1093/molbev/msab015)
Supplement: msab015_Supplementary_Data [file msab015_supplementary_data.pdf]

Supplementary Information to

The Landscape of Recombination Events that Create Nonribosomal Peptide Diversity

Martin Baunach[*,1], Somak Chowdhury[2], Pierre Stallforth[2], Elke Dittmann[1]

[1]University of Potsdam, Institute for Biochemistry and Biology, Karl-Liebknecht-Str. 24/25, 14476 Potsdam-Golm, Germany

[2]Department of Paleobiotechnology, Leibniz Institute for Natural Product Research and Infection Biology Hans Knöll Institute (HKI), Beutenbergstraße 11a, 07745

***Corresponding author:** Martin Baunach; E-mail: baunach@uni-potsdam.de

Content

1. Supplementary tables

Supplementary Table 1. Compounds, genes and proteins analyzed in this study.

2. Supplementary figures

Supplementary FIG. S1. Diversification of NRPs *via* recombination.

Supplementary FIG. S2-S17. Statistical support for recombination event #1-#18.

Supplementary FIG. S18. Subdomain exchange in cyanobacterial NRPS.

Supplementary FIG. S19. Subdomain exchange in non-cyanobacterial NRPS.

Supplementary FIG. S20. Integration of E domains in NRP assembly lines.

Supplementary FIG. S21. Evidence of recombination with horizontally acquired genes in the biosynthesis gene cluster of oscillapeptin E.

Supplementary FIG. S22. Natural exchange units vs. artificial subdomain swaps.

1. Supplementary tables

Supplementary Table S1. Compounds, genes and proteins analyzed in this study.

**Compound**<br>**Investigated producer**	**Accession biosynthesis genes/**<br>**gene clusters**	**Accession**<br>**enzymes**
microcystin-LR<br>*Microcystis aeruginosa* PCC 7806	***mcy***: AF183408(Pearson et al. 2004)<br>BGC0001017 (MIBiG (Kautsar et al. 2020))	**McyB**<br>AAF00962
microcystin-RR<br>*Microcystis aeruginosa* NIES-843	***mcy***: AP009552:3486436-3541027<br>(Kaneko et al. 2007)	**McyB**<br>BAG03678<br>**McyC**<br>BAG03677
[ADMAdda^{5}] microcystin-LR<br>(Sivonen et al. 1992)<br>*Nostoc* sp. 152	***mcyA*** (partial):<br>JQ771631 (Shishido et al. 2013)	**McyA**<br>AGD94570
[D-Leu^{1}] microcystin-LR<br>(Matthiensen et al. 2000)<br>*Nostoc* sp. UK89IIa	***mcyA*** (partial):<br>JX644441 (Shishido et al. 2013)	**McyA**<br>AFW99796
Microginin (Kramer 2006)<br>*Microcystis aeruginosa*	***mic***: DM195384 (Kramer 2006)	Data from Patent<br>(Kramer 2006)
oscillaginin B (Sano and Kaya 1997)<br>*Planktothrix prolifica* NIVA-CYA 98	***mic***: AM990464 (Rounge et al. 2009)	**MicC**<br>CAQ48260<br>**MicD**<br>CAQ48261
anabaenopeptin A<br>(Harada et al. 1995)<br>*Planktothrix prolifica* NIVA-CYA 98	***ana***: AM990463 (Rounge et al. 2009)	**AnaC**<br>CAQ48250<br>**AnaD**<br>CAQ48249
anabaenopeptin 915<br>(Okumura et al. 2009)<br>*Planktothrix agardhii* NIVA-CYA 126/8	***apn***: KU665237 (Entfellner et al. 2017)	**ApnC**<br>AQY60513<br>**ApnD**<br>AQY60514
spumigin A (Fujii et al. 1997)<br>*Nodularia spumigena* CCY9414	***spu***: BGC0000430 (Fewer et al. 2009)<br>(MIBiG)	**SpuB**<br>AHJ31215
speudospumigin A(Jokela et al. 2017)<br>*Nostoc* sp. CENA543	***spu***: MF668123 (Jokela et al. 2017)<br>BGC0001748 (MIBiG)	**SpuB**<br>ATP76246
anabaenolysin A (Jokela et al. 2012)<br>*Anabaena* sp. XSPORK2A	***abl***: KP761742 (Shishido et al. 2015)	**AblD**<br>ALT22120
anabaenolysin C (Shishido et al. 2015)<br>*Anabaena* sp. XPORK13A	***abl***: KP761741 (Shishido et al. 2015)	**AblD**<br>ALT22102
hassallidin A (Neuhof et al. 2005)<br>*Anabaena* sp. Syke748A	***has***: KJ502174 (Vestola et al. 2014)	**HasO**<br>AHZ20774
nostopeptolide A1 (Golakoti et al. 200)<br>*Nostoc* sp. GSV224	***nos***: AF204805.2 (Hoffmann et al. 2003)<br>BGC0001028 (MIBiG)	**NosA**<br>AAF15891.2
cyanopeptolin-984<br>(Tooming-Klunderud et al. 2007)<br>*Microcystis* sp. NIVA-CYA 172/5	***mcn***: DQ075244<br>(Tooming-Klunderud et al. 2007)	**McnC**<br>AAZ03552<br>**McnE**<br>AAZ03554
oscillapeptin E (Itou et al. 1999)<br>*Planktothrix agardhii* NIES 205	***oci***: EU109504 (Rounge et al. 2008)	**OciA**<br>ABW84363

		**OciE**<br>ABW84364
oscillapeptin G (Sano and Kaya 1996)<br>*Planktothrix rubescens* NIVA-CYA 98	***oci***: AM990463.2 (Rounge et al. 2009)	**OciB**<br>CAQ48255<br>**OciC**<br>CAQ48258
cyanopeptolin 963A<br>(Bister et al. 2004)<br>*Microcystis aeruginosa* PCC 7806	***mcn***: AM778942: 63976-93086	**McnC**<br>CAO90637<br>**McnE**<br>Self-annotated
micropeptin 88-A (Ishida et al. 1998)<br>*Microcystis aeruginosa* NIES-88	***mcn***: JXYX01000001: 396006- 431374	**McnC**<br>KXS92360<br>**McnE**<br>KXS92361
micropeptin K139 (Harada et al. 2004)<br>*Microcystis aeruginosa* K-139	***mcn***: AB481215 (Nishizawa et al. 2011)<br>BGC0001018 (MIBiG)	**McnC**<br>BAH22764<br>**McnE**<br>BAH22765
nostopeptin E (Sun et al. 1998)<br>*Nostoc linckia* NIES-25	***oci***: AP018222: c5817676- 5808142	**OciAB**<br>BAY78314
nostopeptin B (Okino et al. 1997)<br>*Nostoc minutum* NIES-26	***oci***: LXQD01000316: 1878-34182	**OciAB**<br>RCJ25078
cyanopeptolin 1138<br>(Rounge et al. 2007)<br>*Planktothrix agardhii* NIVA-CYA 116	***oci***: DQ837301 (Rounge et al. 2007)	**OciA**<br>ABI26077
Nostophycin (Fujii et al. 1999)<br>*Nostoc* sp. 152	***npn***: JF430079 (Fewer et al. 2011)<br>BGC0001029 (MIBiG)	**NpnB**<br>AEU11006
Mycosubtilin (Peypoux et al. 1986)<br>*Bacillus subtilis* ATCC 6633	***myc***: AF184956 (Duitman et al. 1999)<br>BGC0001103 (MIBiG)	**MycB**<br>AAF08796<br>**MycC**<br>AAF08797
mojavensin A (Ma et al. 2012)<br>*Bacillus tequilensis* NCTC 13306	***ituB***: UAQB01000027:c198053-181959<br>(Dunlap et al. 2019)<br>***ituC***: AQB01000027:174053-181870<br>(Dunlap et al. 2019)	**ItuB**<br>SPT99259<br>**ItuC**<br>SPT99257
iturin A (Peypoux et al. 1978)<br>*Bacillus subtilis* RB14	***itu***: AB050629 (Tsuge et al. 2001)<br>BGC0001098 (MIBiG)	**ItuB**<br>BAB69699<br>**ItuC**<br>BAB69700
bacillomycin F (Peypoux et al. 1985)<br>*Bacillus subtilis* KCTC 13429	***bmyB***: CP029465:2095894-2111987<br>(Dunlap et al. 2019)<br>***bmyC***: CP029465:2087978-2095804<br>(Dunlap et al. 2019)	**BmyB**<br>Self-annotated<br>**BmyC**<br>AWM17129
bacillomycin D(Peypoux et al. 1984)<br>*Bacillus velezensis* FZB42	***bmy***: BGC0001090<br>(Koumoutsi et al. 2004) (MIBiG)	**BmyB**<br>ABS74180
bacillomycin L (Volpon et al. 2007)<br>*Bacillus velezensis* KACC 18228	***bmyB***: LLZA01000001.1:44524-60651<br>(Dunlap et al. 2019)	**BmyB**<br>KSW05789
D-Dab$_3$-polymyxin B$_1$<br>(Shaheen et al. 2011)<br>*Paenibacillus polymyxia* PKB1	***pxm***: JN660148 (Shaheen et al. 2011)	**PxmA**<br>AEZ51516
polymyxin P$_1$<br>(Kimura et al. 1969)<br>*Paenibacillus polymyxa* M1	***pxm***: FR727736 (Niu et al. 2013)	**PxmA**<br>CBY05531

polymyxin E_1 (Wilkinson and Lowe 1964) *Paenibacillus polymyxia* ATCC21830	Data from patent (Park S-H et al. 2012)	Data from patent (Park S-H et al. 2012)
polymyxin A_1 (Wilkinson and Lowe 1966) *Paenibacillus polymyxia* E681	***pxm***: EU371992 (Choi et al. 2009) BGC0000408 (MIBiG)	**PxmA** ACA97576
D-Dab_3-polymyxin E_1 * (Tambadou et al. 2015) *Paenibacillus alvei*	***pxm***: KP262070 (Tambadou et al. 2015) BGC0001192 (MIBiG)	**PxmE** AJM89738
fusaricidin A(Kajimura and Kaneda 1996) *Paenibacillus polymyxa* PKB1	***fus***: EF451155.3 (Li et al. 2007) BGC0001152 (MIBiG)	**FusA** ABQ96384.2
Fengycin (Vanittanakom et al. 1986) *Bacillus velezensis* FZB42	***fen***: AJ576102 (Koumoutsi et al. 2004) BGC0001095 (MIBiG)	**FenA** CAE11274
Vancomycin (Williams and Kalman 1977) *Amycolatopsis orientalis* HCCB10007	***vcm***: HQ679900 (Xu et al. 2014) BGC0000455 (MIBiG)	**Vcm2** AEI58865
Pekiskomycin (Thaker et al. 2013) *Streptomyces* sp. WAC1420	***pek***: JX026280 (Thaker et al. 2013)	**Pek18** AGF91753
aeruginoside 126A (Ishida et al. 2007) *Planktothrix agardhii* NIVA-CYA 126/8	***aer***: AM071396 (Ishida et al. 2007) BGC0000297 (MIBiG)	**AerB** CAJ21198.2
aeruginosin A (Rounge et al. 2009) *Planktothrix prolifica* NIVA-CYA 98	***aer***: AM990465 (Rounge et al. 2009)	**AerB** CAQ48266
aeruginosin 98A (Murakami et al. 1995) *Microcystis aeruginosa* NIES-98	***aer***: FJ609416 (Ishida et al. 2009) BGC0000298 (MIBiG)	**AerB** ACM68684
aeruginosin 102A (Matsuda et al. 1996) *Microcystis aeruginosa* NIES-102	***aer***: AP019314:c3064789-3083819	**AerB** BBH40328
Hormaomycin (Zlatopolskiy et al. 2004) *Streptomyces griseoflavus* W-384	***hrm***: HQ542230 (Höfer et al. 2011) BGC0000374 (MIBiG)	**HrmO** AEH41793

*The stereochemistry of Dab in position 3 of D-Dab_3-polymyxin E_1 has not been validated experimentally but is assumed based on the presence of an epimerase domain (Tambadou et al. 2015).

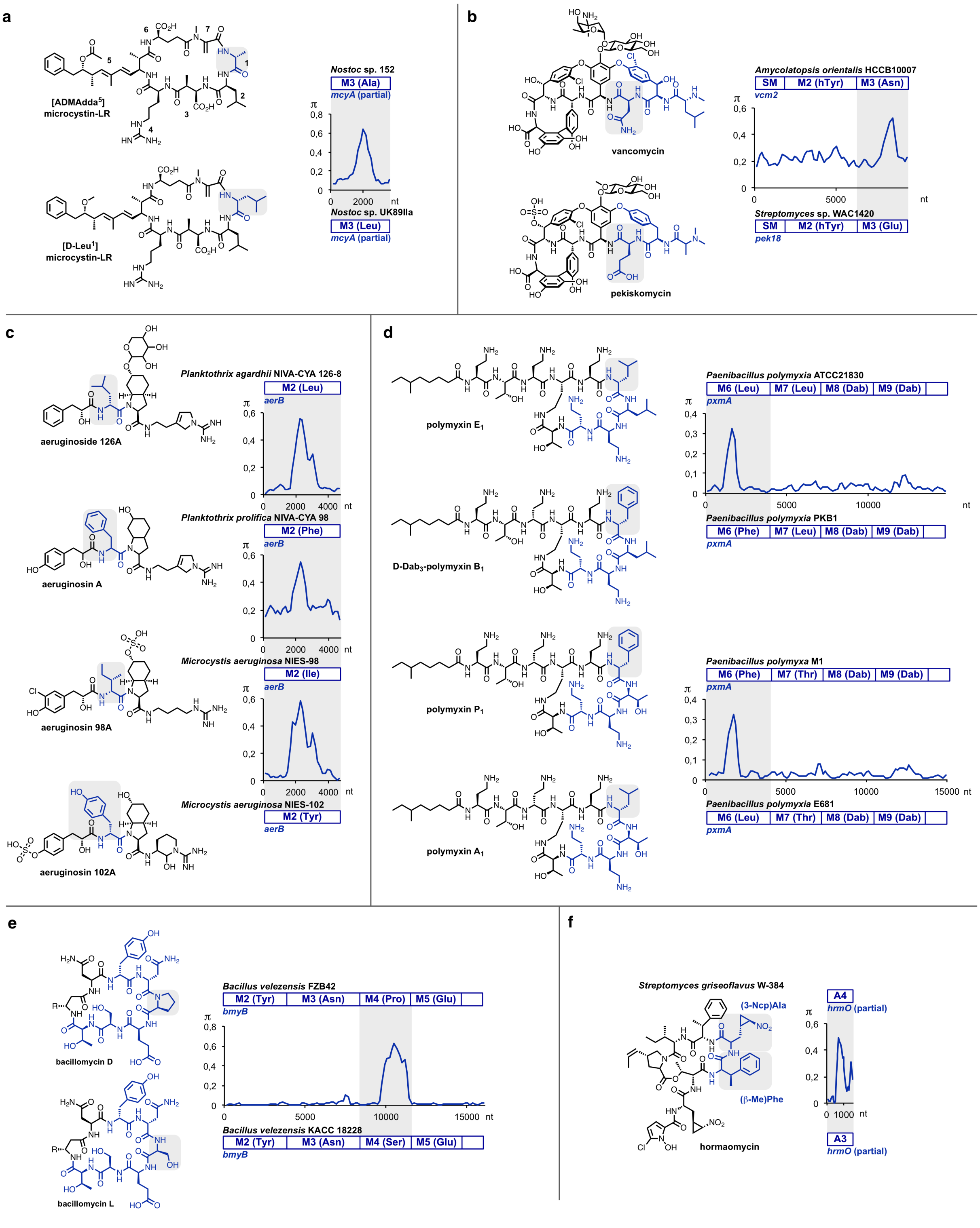

Supplementary FIG. S1. Diversification of NRPs via recombination. Examples for the diversification of bacterial NRPs *via* recombination from the biosynthesis of **a**, microcystins, **b**, glycopeptide antibiotics, **c**, aeruginosins, **d**, polymyxins, **e**, iturinic lipopeptides, and **f**, hormaomycin, for which no plausible recombination partner sequences from characterized NRP biosynthesis genes could be identified. Structural differences of compound pairs (grey squares) correlate with nucleotide sequence polymorphisms of the genes encoding NRPS modules (M), thereby indicating recombination. Most closely related sequences have been aligned for pairwise comparison. π values (average number of nucleotide differences per site between two sequences) were computed in the sliding window mode in DnaSP (width, 300 nt; step, 150 nt). Amino acid residues in the structures are color-coded to trace back their biosynthetic origin to individual modules. Dab, diaminobutyric acid; (3-Ncp)Ala, 3-nitrocyclopropylalanine; R, alkyl moiety.

a

①

			Breakpoint Positions in Recombinant					Detection Methods				
Recombinant	Minor Parent	Major Parent	Begin	End	RDP	GENECONV	Bootscan	Maxchi	Chimaera	SiSscan	3Seq	LARD
M4_mycB(Arg)	M6_mycB(Arg)	M4_mycB(Leu)	1850	2735	4,30E-136	2,98E-145	1,32E-131	1,71E-44	1,39E-36	2,30E-53	1,11E-16	1,19E-201

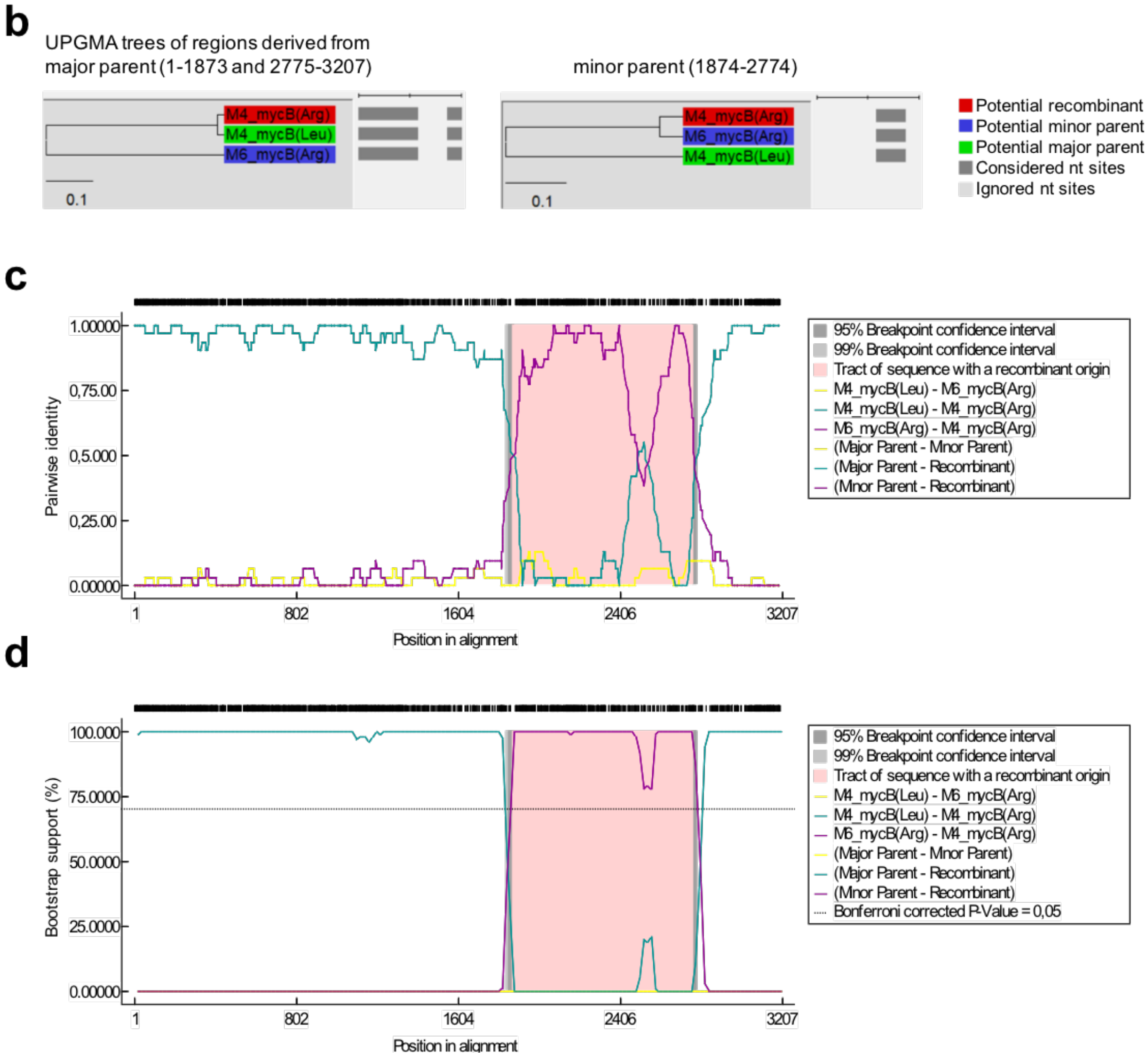

Supplementary FIG. S2. Statistical support for recombination event #1 by using Recombination Detection Program version 4 (RDP4) (Martin et al. 2015). **a**, Probability scores for recombination calculated by the recombination detection methods RDP (Martin and Rybicki 2000), GENECONV (Padidam et al. 1999), Bootscan (Salminen et al. 1995), Maxchi (Smith 1992), Chimaera (Posada and Crandall 2001), SiSscan (Gibbs et al. 2000), 3Seq (Boni et al. 2007) and LARD (Holmes et al. 1999) together with the prediction of the recombinant as well as the major and minor parent. RDP4 warns about a possible misidentification of the recombinant. However, the assignment seems logical. **b**, 'Recombination aware' phylogenetic trees of the putative recombination event. **c**, Graphical output of recombination detection method RDP. **d**, Graphical output of recombination detection method Bootscan.

a

(2)

Recombinant	Minor Parent	Major Parent	Breakpoint Positions in Recombinant: Begin	End	Detection Methods: RDP	GENECONV	Bootscan	Maxchi	Chimaera	SiSscan	3Seq	LARD
M2_micC(Val)	M7_mcnE(Val)	M2_micC(Leu)	1369	2633	1,64E-100	9,70E-73	3,65E-66	5,40E-45	8,10E-35	3,42E-50	4,44E-16	3,14E-116

b UPGMA trees of regions derived from major parent (1-1383 and 2775-2679) minor parent (1384-2678)

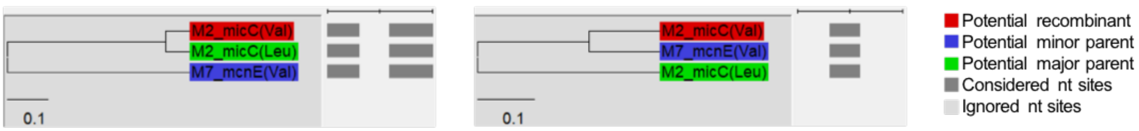

c

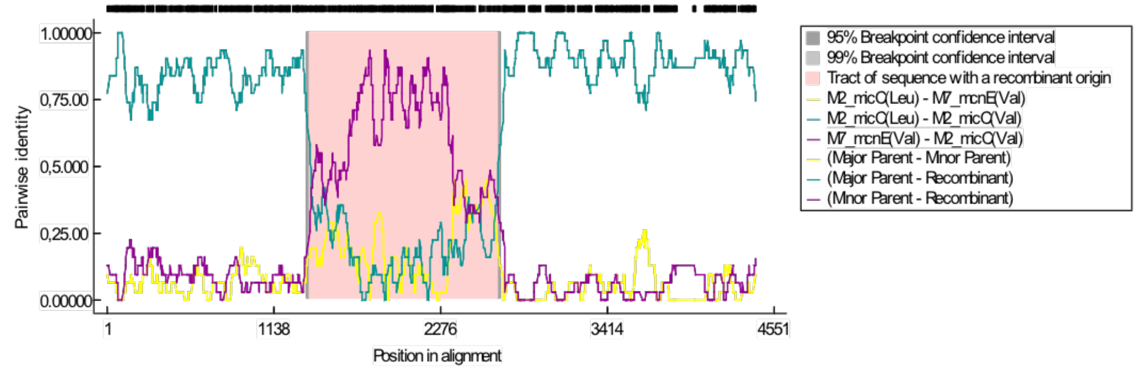

d

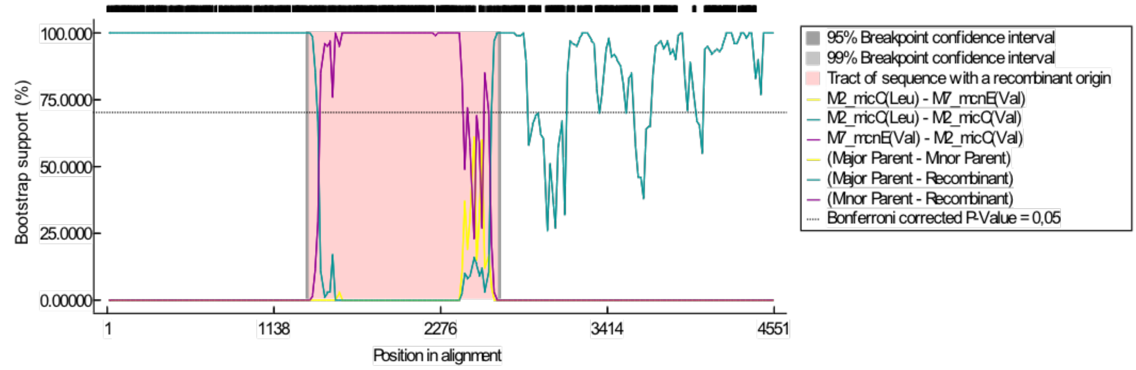

Supplementary FIG. S3. Statistical support for recombination event #2 by using Recombination Detection Program version 4 (RDP4) (Martin et al. 2015). **a**, Probability scores for recombination calculated by the recombination detection methods RDP (Martin and Rybicki 2000), GENECONV (Padidam et al. 1999), Bootscan (Salminen et al. 1995), Maxchi (Smith 1992), Chimaera (Posada and Crandall 2001), SiSscan (Gibbs et al. 2000), 3Seq (Boni et al. 2007) and LARD (Holmes et al. 1999) together with the prediction of the recombinant as well as the major and minor parent. RDP4 warns about a possible misidentification of the recombinant. However, the assignment seems logical. **b**, 'Recombination aware' phylogenetic trees of the putative recombination event. **c**, Graphical output of recombination detection method RDP. **d**, Graphical output of recombination detection method Bootscan.

a

③

			Breakpoint Positions in Recombinant				Detection Methods					
Recombinant	Minor Parent	Major Parent	Begin	End	RDP	GENECONV	Bootscan	Maxchi	Chimaera	SiSscan	3Seq	LARD
M3_micD(Hty)	M4_anaC(Hty)	M3_micD(Tyr)	1789	2540	8,71E-71	1,90E-67	1,28E-69	2,52E-30	7,05E-26	5,08E-47	1,11E-16	3,37E-58

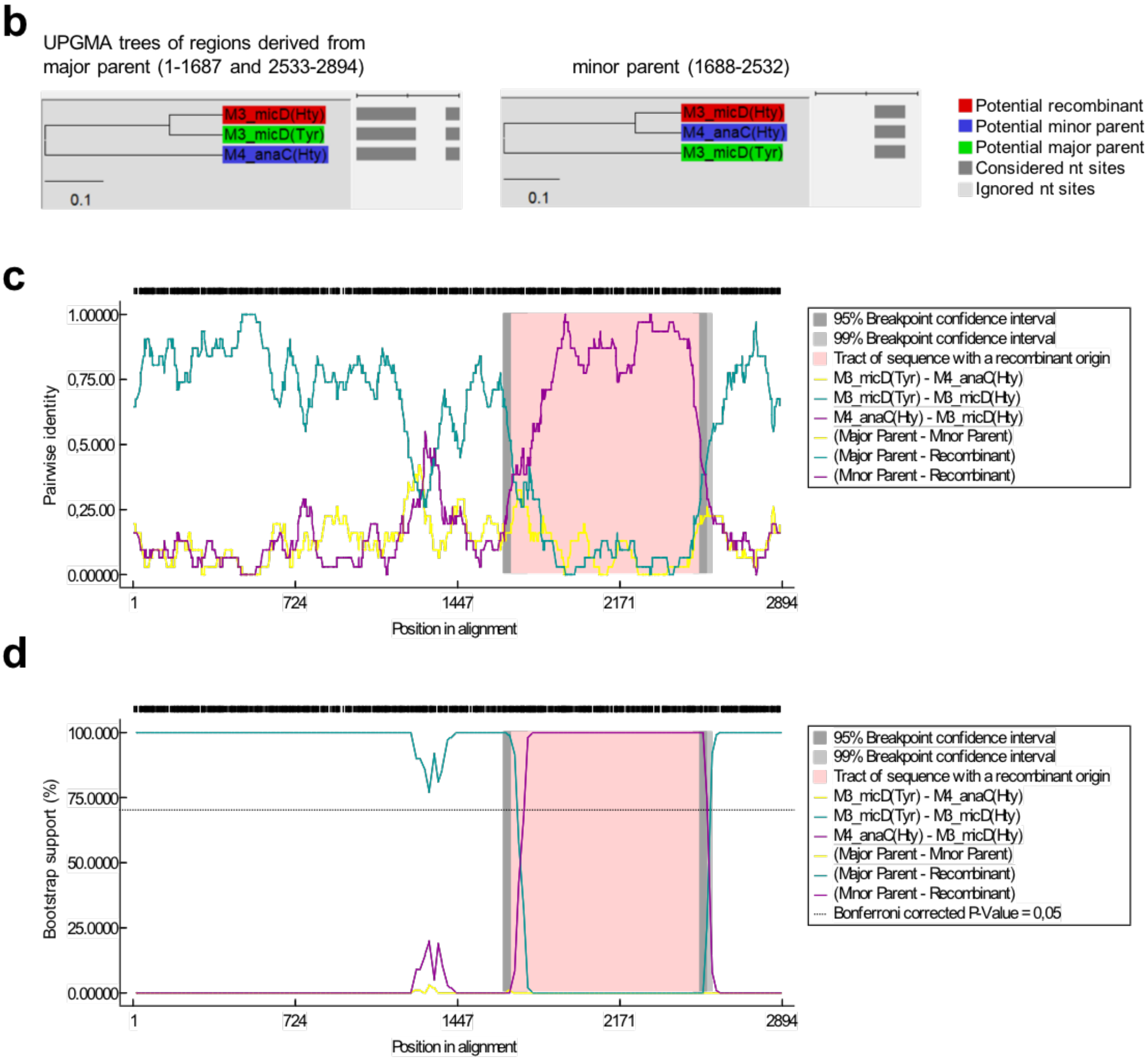

Supplementary FIG. S4. Statistical support for recombination event #3 by using Recombination Detection Program version 4 (RDP4) (Martin et al. 2015). **a**, Probability scores for recombination calculated by the recombination detection methods RDP (Martin and Rybicki 2000), GENECONV (Padidam et al. 1999), Bootscan (Salminen et al. 1995), Maxchi (Smith 1992), Chimaera (Posada and Crandall 2001), SiSscan (Gibbs et al. 2000), 3Seq (Boni et al. 2007) and LARD (Holmes et al. 1999) together with the prediction of the recombinant as well as the major and minor parent. RDP4 warns about a possible misidentification of the recombinant. However, the assignment seems logical. **b**, 'Recombination aware' phylogenetic trees of the putative recombination event. **c**, Graphical output of recombination detection method RDP. **d**, Graphical output of recombination detection method Bootscan.

a

			Breakpoint Positions in Recombinant		Detection Methods							
Recombinant	Minor Parent	Major Parent	Begin	End	RDP	GENECONV	Bootscan	Maxchi	Chimaera	SiSscan	3Seq	LARD
⑤ M3_spuB(Ile)	M1_nosA(Ile)	M3_spuB(mPro)	1640	2860	1,43E-13	2,47E-08	3,25E-12	1,74E-12	6,06E-04	2,11E-41	7,30E-16	3,40E-21

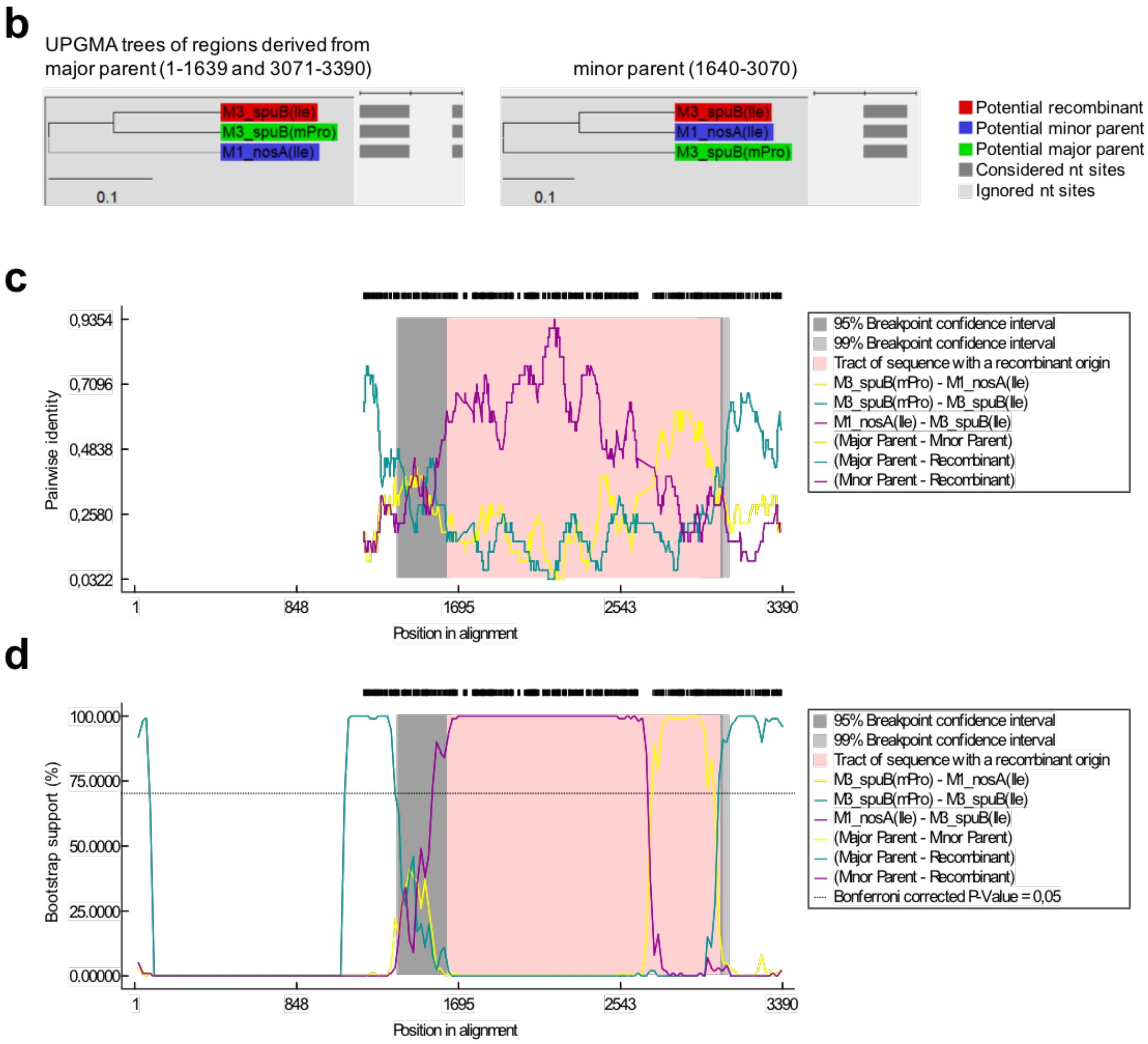

Supplementary FIG. S5. Statistical support for recombination event #5 by using Recombination Detection Program version 4 (RDP4) (Martin et al. 2015). **a**, Probability scores for recombination calculated by the recombination detection methods RDP (Martin and Rybicki 2000), GENECONV (Padidam et al. 1999), Bootscan (Salminen et al. 1995), Maxchi (Smith 1992), Chimaera (Posada and Crandall 2001), SiSscan (Gibbs et al. 2000), 3Seq (Boni et al. 2007) and LARD (Holmes et al. 1999) together with the prediction of the recombinant as well as the major and minor parent. RDP4 warns about a possible misidentification of the recombinant. However, the assignment seems logical. **b**, 'Recombination aware' phylogenetic trees of the putative recombination event. **c**, Graphical output of recombination detection method RDP. **d**, Graphical output of recombination detection method Bootscan.

a

	Recombinant	Minor Parent	Major Parent	Breakpoint Positions in Recombinant: Begin	End	RDP	GENECONV	Bootscan	Detection Methods: Maxchi	Chimaera	SiSscan	3Seq	LARD
⑥	M2_ablD(Gln)	M6_hasO(Gln)	M2_ablD(Gly)	1614	2319	3,00E-89	3,60E-63	7,01E-74	4,71E-30	8,31E-34	2,18E-43	3,33E-16	4,28E-63

b

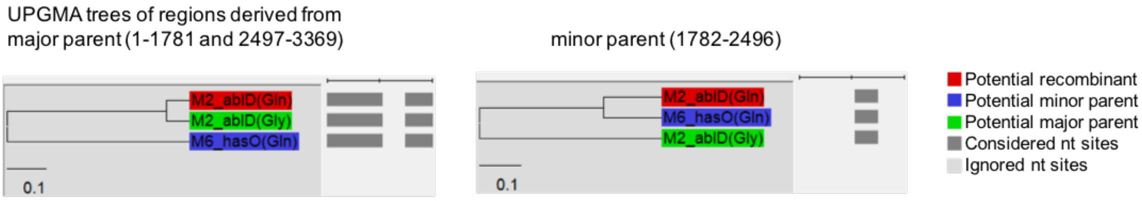

c

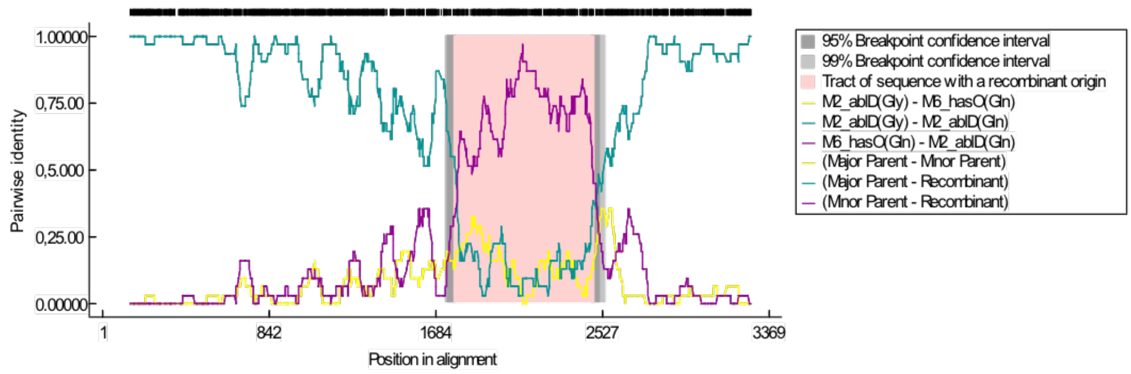

d

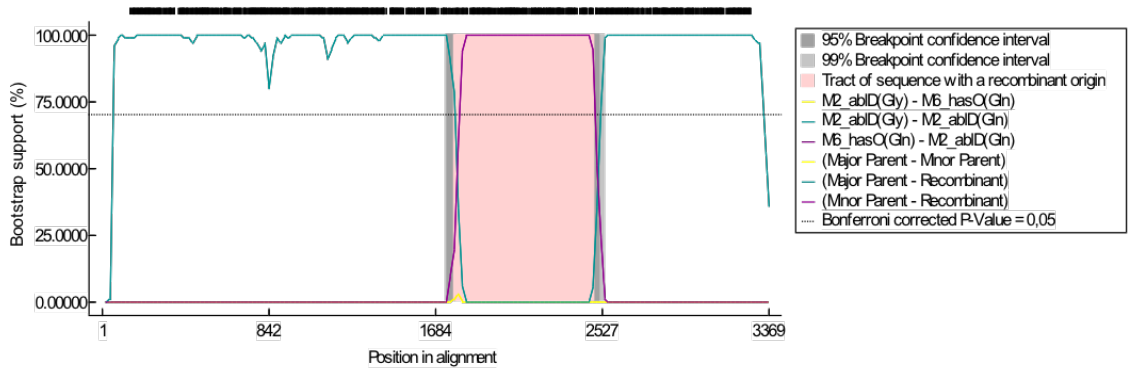

Supplementary FIG. S6. Statistical support for recombination event #6 by using Recombination Detection Program version 4 (RDP4) (Martin et al. 2015). **a**, Probability scores for recombination calculated by the recombination detection methods RDP (Martin and Rybicki 2000), GENECONV (Padidam et al. 1999), Bootscan (Salminen et al. 1995), Maxchi (Smith 1992), Chimaera (Posada and Crandall 2001), SiSscan (Gibbs et al. 2000), 3Seq (Boni et al. 2007) and LARD (Holmes et al. 1999) together with the prediction of the recombinant as well as the major and minor parent. RDP4 warns about a possible misidentification of the recombinant. However, the assignment seems logical. **b**, 'Recombination aware' phylogenetic trees of the putative recombination event. **c**, Graphical output of recombination detection method RDP. **d**, Graphical output of recombination detection method Bootscan.

a

				Breakpoint Positions in Recombinant		Detection Methods							
	Recombinant	Minor Parent	Major Parent	Begin	End	RDP	GENECONV	Bootscan	Maxchi	Chimaera	SiSscan	3Seq	LARD
⑦	M5_mcnC(Phe)	M7_mcnE(Ile)	M5_ociB(Ile)	1576	2465	8,19E-150	7,12E-154	6,60E-163	2,16E-48	5,93E-34	9,17E-65	1,11E-16	6,29E-126

b

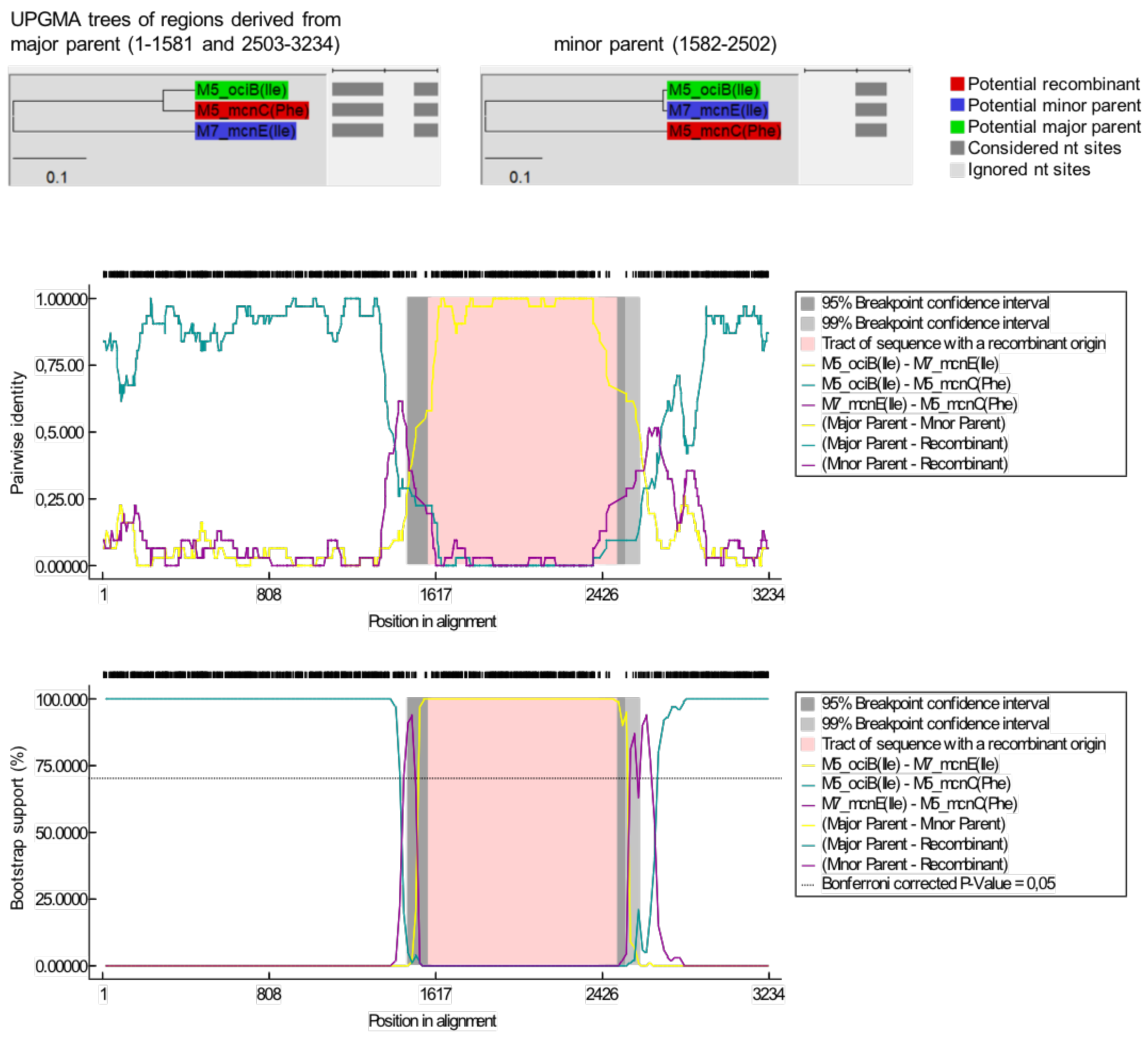

Supplementary FIG. S7. Statistical support for recombination event #7 by using Recombination Detection Program version 4 (RDP4) (Martin et al. 2015). **a**, Probability scores for recombination calculated by the recombination detection methods RDP (Martin and Rybicki 2000), GENECONV (Padidam et al. 1999), Bootscan (Salminen et al. 1995), Maxchi (Smith 1992), Chimaera (Posada and Crandall 2001), SiSscan (Gibbs et al. 2000), 3Seq (Boni et al. 2007) and LARD (Holmes et al. 1999) together with the prediction of the recombinant as well as the major and minor parent. RDP4 warns about a possible misidentification of the recombinant and states that M5_ociB(Ile) or M7_mcnE(Ile) may be the actual recombinant. Since the assignment of M5_mcnC(Phe) as the recombinant does not make sense from a biosynthetic perspective we suggest M5_ociB(Ile) to be the recombinant. **b**, ‘Recombination aware’ phylogenetic trees of the putative recombination event. **c**, Graphical output of recombination detection method RDP. **d**, Graphical output of recombination detection method Bootscan.

a

⑧

			Breakpoint Positions in Recombinant		Detection Methods							
Recombinant	Minor Parent	Major Parent	Begin	End	RDP	GENECONV	Bootscan	Maxchi	Chimaera	SiSscan	3Seq	LARD
M6_ociB(Thr)	M3_ociA(Thr)	M5_ociB(Ile)	1657	2490	3,07E-99	8,57E-92	2,85E-88	1,47E-42	2,51E-27	3,06E-50	1,11E-16	1,61E-92

b

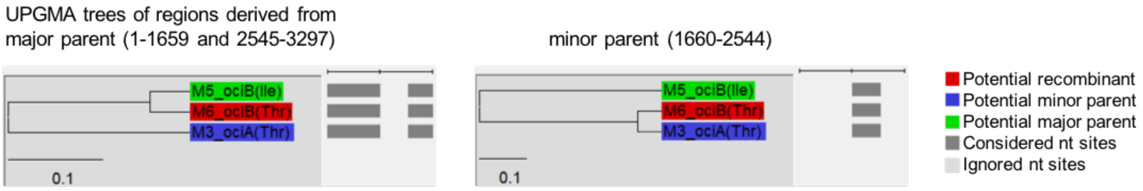

c

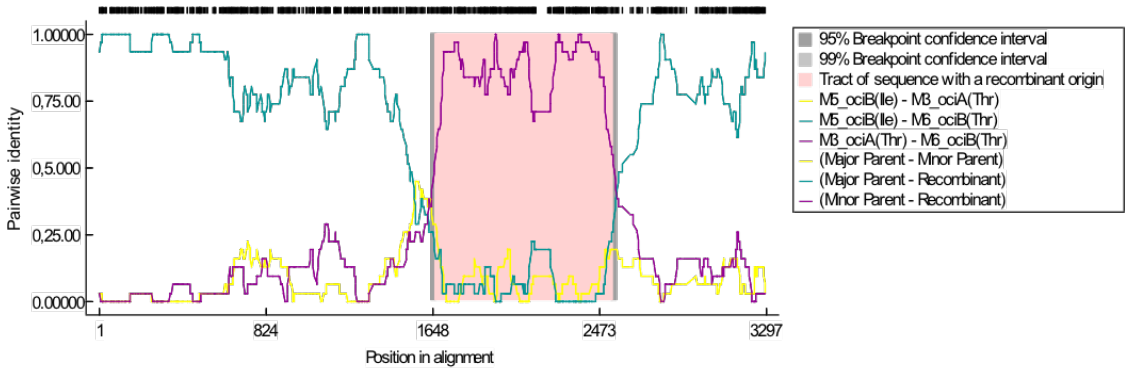

d

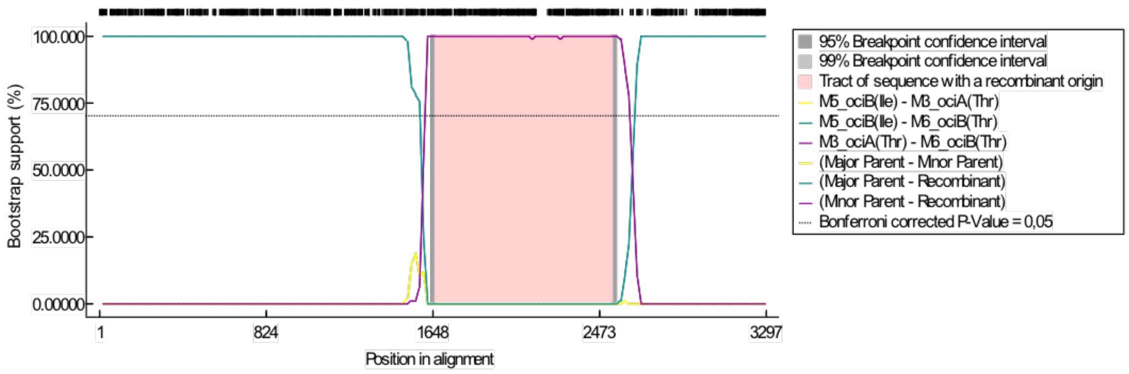

Supplementary FIG. S8. Statistical support for recombination event #8 by using Recombination Detection Program version 4 (RDP4) (Martin et al. 2015). **a**, Probability scores for recombination calculated by the recombination detection methods RDP (Martin and Rybicki 2000), GENECONV (Padidam et al. 1999), Bootscan (Salminen et al. 1995), Maxchi (Smith 1992), Chimaera (Posada and Crandall 2001), SiSscan (Gibbs et al. 2000), 3Seq (Boni et al. 2007) and LARD (Holmes et al. 1999) together with the prediction of the recombinant as well as the major and minor parent. RDP4 warns about a possible misidentification of the recombinant. However, the assignment seems logical. **b**, 'Recombination aware' phylogenetic trees of the putative recombination event. **c**, Graphical output of recombination detection method RDP. **d**, Graphical output of recombination detection method Bootscan.

a

9

			Breakpoint Positions in Recombinant					Detection Methods				
Recombinant	Minor Parent	Major Parent	Begin	End	RDP	GENECONV	Bootscan	Maxchi	Chimaera	SiSscan	3Seq	LARD
M7_mcnE(Val)	M2_micC(Val)	M8_ociC(Ile)	1870	2381	9,62E-50	1,59E-41	1,48E-43	4,45E-22	1,34E-18	2,43E-35	4,70E-27	2,47E-45

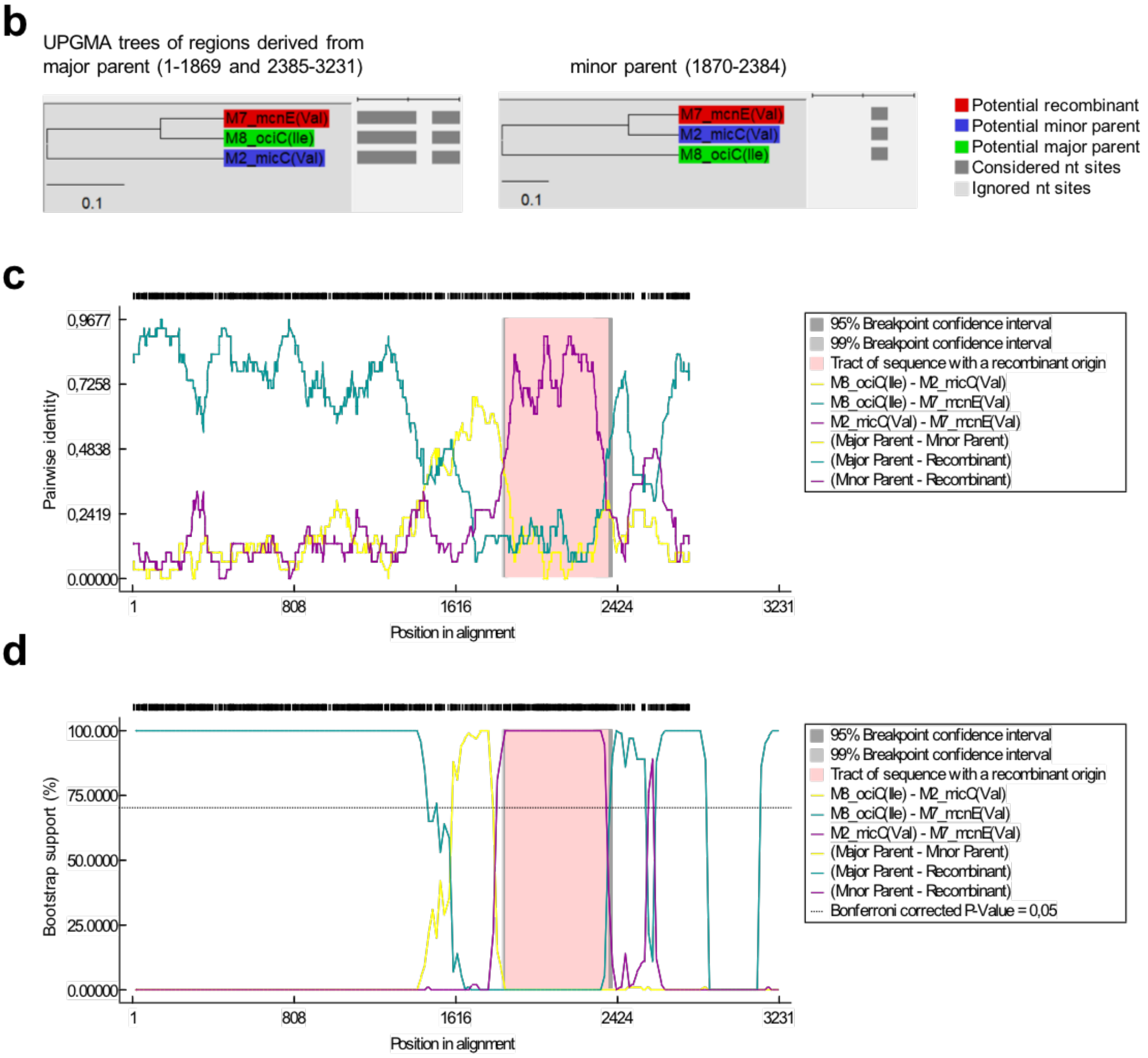

Supplementary FIG. S9. Statistical support for recombination event #9 by using Recombination Detection Program version 4 (RDP4) (Martin et al. 2015). **a**, Probability scores for recombination calculated by the recombination detection methods RDP (Martin and Rybicki 2000), GENECONV (Padidam et al. 1999), Bootscan (Salminen et al. 1995), Maxchi (Smith 1992), Chimaera (Posada and Crandall 2001), SiSscan (Gibbs et al. 2000), 3Seq (Boni et al. 2007) and LARD (Holmes et al. 1999) together with the prediction of the recombinant as well as the major and minor parent. RDP4 warns about a possible misidentification of the recombinant. However, the assignment seems logical. **b**, 'Recombination aware' phylogenetic trees of the putative recombination event. **c**, Graphical output of recombination detection method RDP. **d**, Graphical output of recombination detection method Bootscan.

a

10

			Breakpoint Positions in Recombinant					Detection Methods				
Recombinant	Minor Parent	Major Parent	Begin	End	RDP	GENECONV	Bootscan	Maxchi	Chimaera	SiSscan	3Seq	LARD
M6_mcnC(Val)	M7_mcnC(Val)	M5_mcnC(Ile)	1620	2457	9,62E-110	1,10E-122	2,84E-122	2,24E-39	1,69E-31	8,53E-53	6,00E-35	5,77E-84

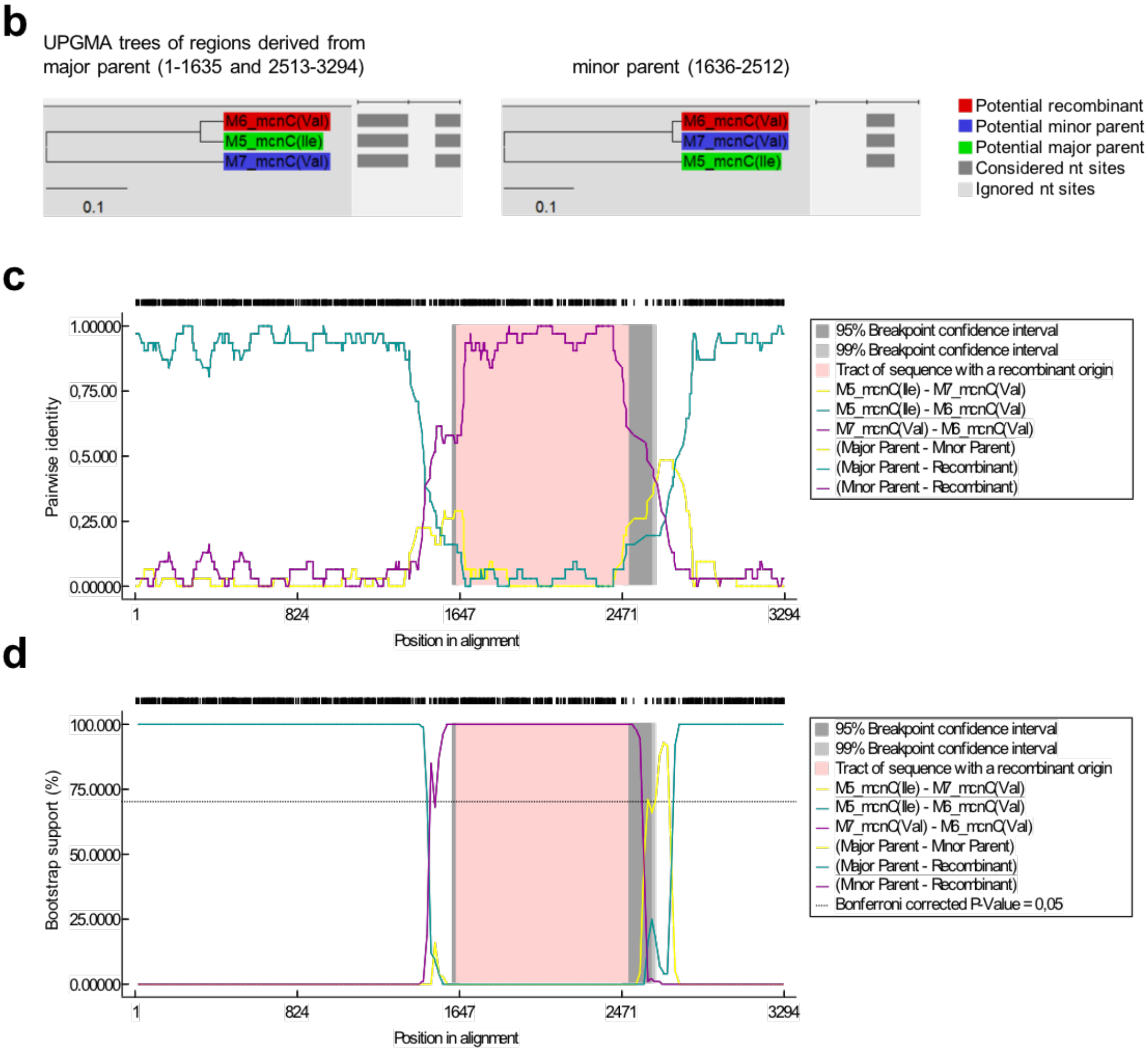

Supplementary FIG. S10. Statistical support for recombination event #10 by using Recombination Detection Program version 4 (RDP4) (Martin et al. 2015). **a**, Probability scores for recombination calculated by the recombination detection methods RDP (Martin and Rybicki 2000), GENECONV (Padidam et al. 1999), Bootscan (Salminen et al. 1995), Maxchi (Smith 1992), Chimaera (Posada and Crandall 2001), SiSscan (Gibbs et al. 2000), 3Seq (Boni et al. 2007) and LARD (Holmes et al. 1999) together with the prediction of the recombinant as well as the major and minor parent. RDP4 warns about a possible misidentification of the recombinant. However, the assignment seems logical. **b**, 'Recombination aware' phylogenetic trees of the putative recombination event. **c**, Graphical output of recombination detection method RDP. **d**, Graphical output of recombination detection method Bootscan.

a

11

			Breakpoint Positions in Recombinant					Detection Methods				
Recombinant	Minor Parent	Major Parent	Begin	End	RDP	GENECONV	Bootscan	Maxchi	Chimaera	SiSscan	3Seq	LARD
M6_mcnC(Val)	M7_McnE(Ile)	M5_mcnC(Ile)	1389	2431	9,13E-143	4,38E-143	1,19E-149	1,46E-41	5,71E-42	1,08E-53	2,22E-16	9,01E-123

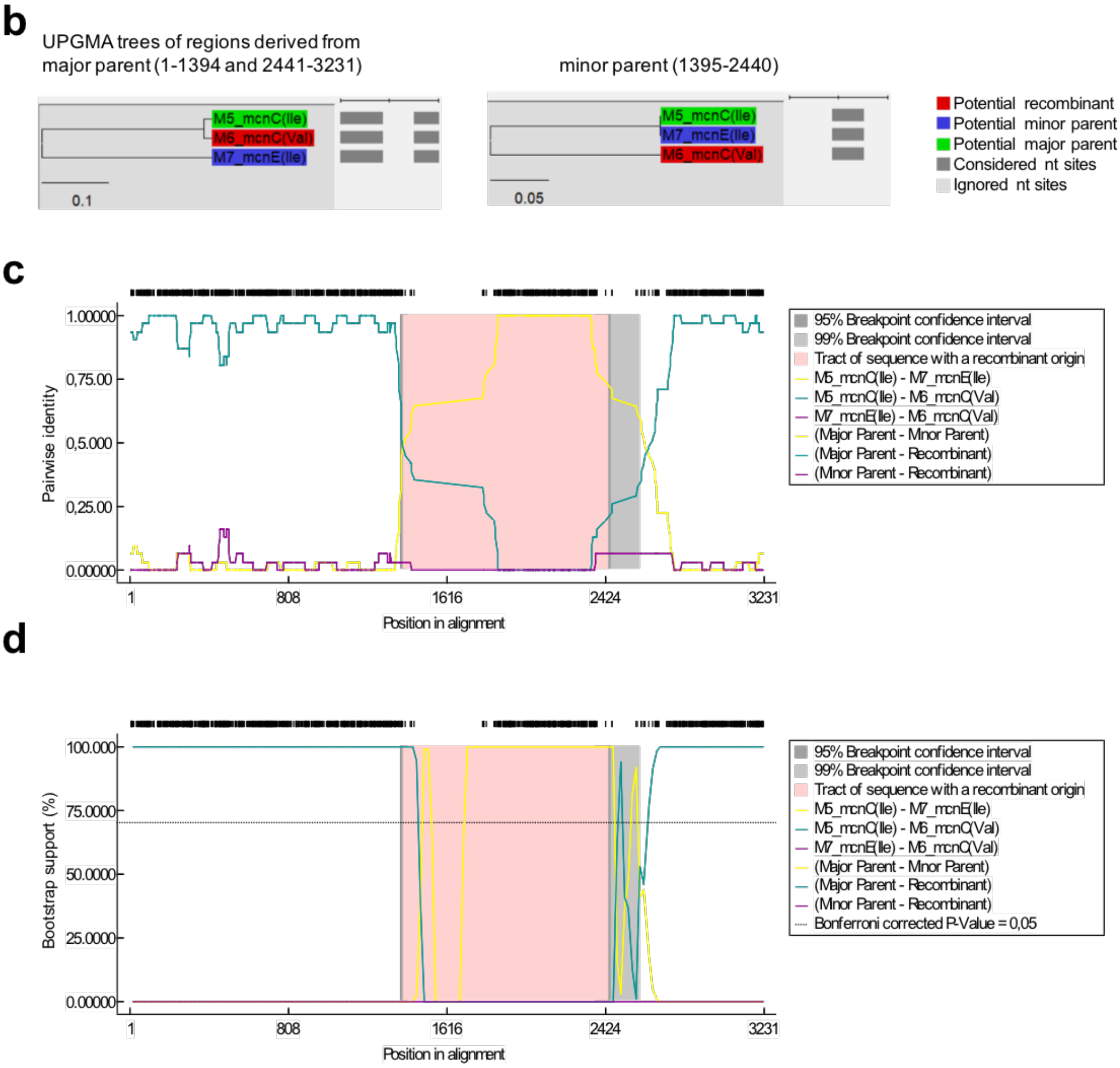

Supplementary FIG. S11. Statistical support for recombination event #11 by using Recombination Detection Program version 4 (RDP4) (Martin et al. 2015). **a**, Probability scores for recombination calculated by the recombination detection methods RDP (Martin and Rybicki 2000), GENECONV (Padidam et al. 1999), Bootscan (Salminen et al. 1995), Maxchi (Smith 1992), Chimaera (Posada and Crandall 2001), SiSscan (Gibbs et al. 2000), 3Seq (Boni et al. 2007) and LARD (Holmes et al. 1999) together with the prediction of the recombinant as well as the major and minor parent. RDP4 warns about a possible misidentification of the recombinant and states that M5_mcnC(Ile) or M7_mcnE(Ile) may be the actual recombinant. Since the assignment of M6_mcnC(Val) as the recombinant does not make sense from a biosynthetic perspective we suggest M5_mcnC(Ile) to be the recombinant. **b**, 'Recombination aware' phylogenetic trees of the putative recombination event. **c**, Graphical output of recombination detection method RDP. **d**, Graphical output of recombination detection method Bootscan.

a

12

			Breakpoint Positions in Recombinant				Detection Methods					
Recombinant	Minor Parent	Major Parent	Begin	End	RDP	GENECONV	Bootscan	Maxchi	Chimaera	SiSscan	3Seq	LARD
M1_ociAB(Gln)	M1_npnB(Gln)	M1_ociAB(Arg)	1876	2598	4,29E-71	5,71E-61	9,10E-57	6,00E-24	1,13E-32	1,51E-40	3,33E-16	5,90E-79

b

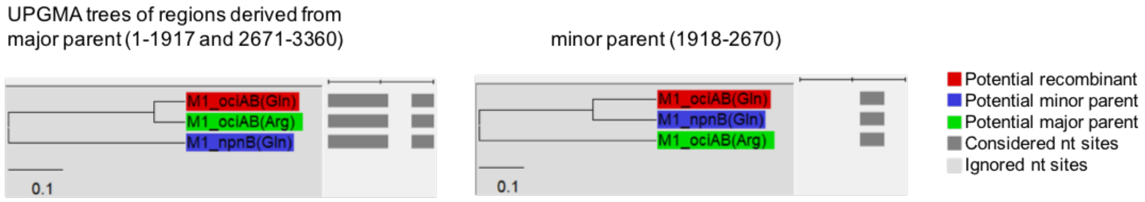

c

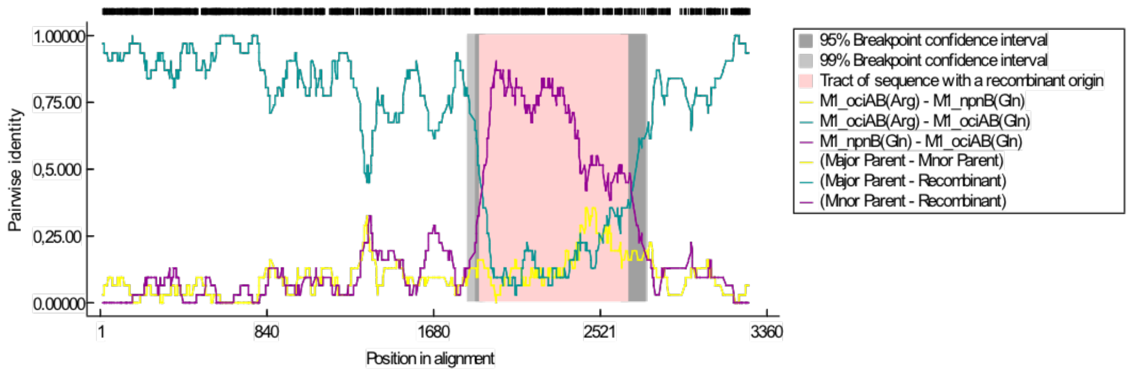

d

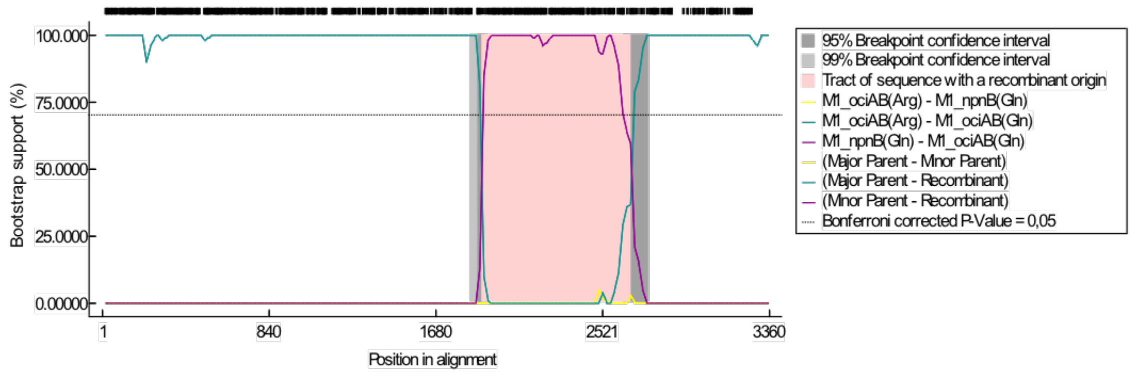

Supplementary FIG. S12. Statistical support for recombination event #12 by using Recombination Detection Program version 4 (RDP4) (Martin et al. 2015). **a**, Probability scores for recombination calculated by the recombination detection methods RDP (Martin and Rybicki 2000), GENECONV (Padidam et al. 1999), Bootscan (Salminen et al. 1995), Maxchi (Smith 1992), Chimaera (Posada and Crandall 2001), SiSscan (Gibbs et al. 2000), 3Seq (Boni et al. 2007) and LARD (Holmes et al. 1999) together with the prediction of the recombinant as well as the major and minor parent. RDP4 warns about a possible misidentification of the recombinant. However, the assignment seems logical. **b**, 'Recombination aware' phylogenetic trees of the putative recombination event. **c**, Graphical output of recombination detection method RDP. **d**, Graphical output of recombination detection method Bootscan.

a

13

Recombinant	Minor Parent	Major Parent	Breakpoint Positions in Recombinant Begin	End	Detection Methods RDP	GENECONV	Bootscan	Maxchi	Chimaera	SiSscan	3Seq	LARD
M5_ociAB(Ile)	M5_ociAB(Leu)	M7_ociAB(Ile)	2836	1409	4,47E-72	1,70E-78	3,55E-81	6,15E-48	1,71E-26	9,48E-79	1,11E-16	3,77E-119

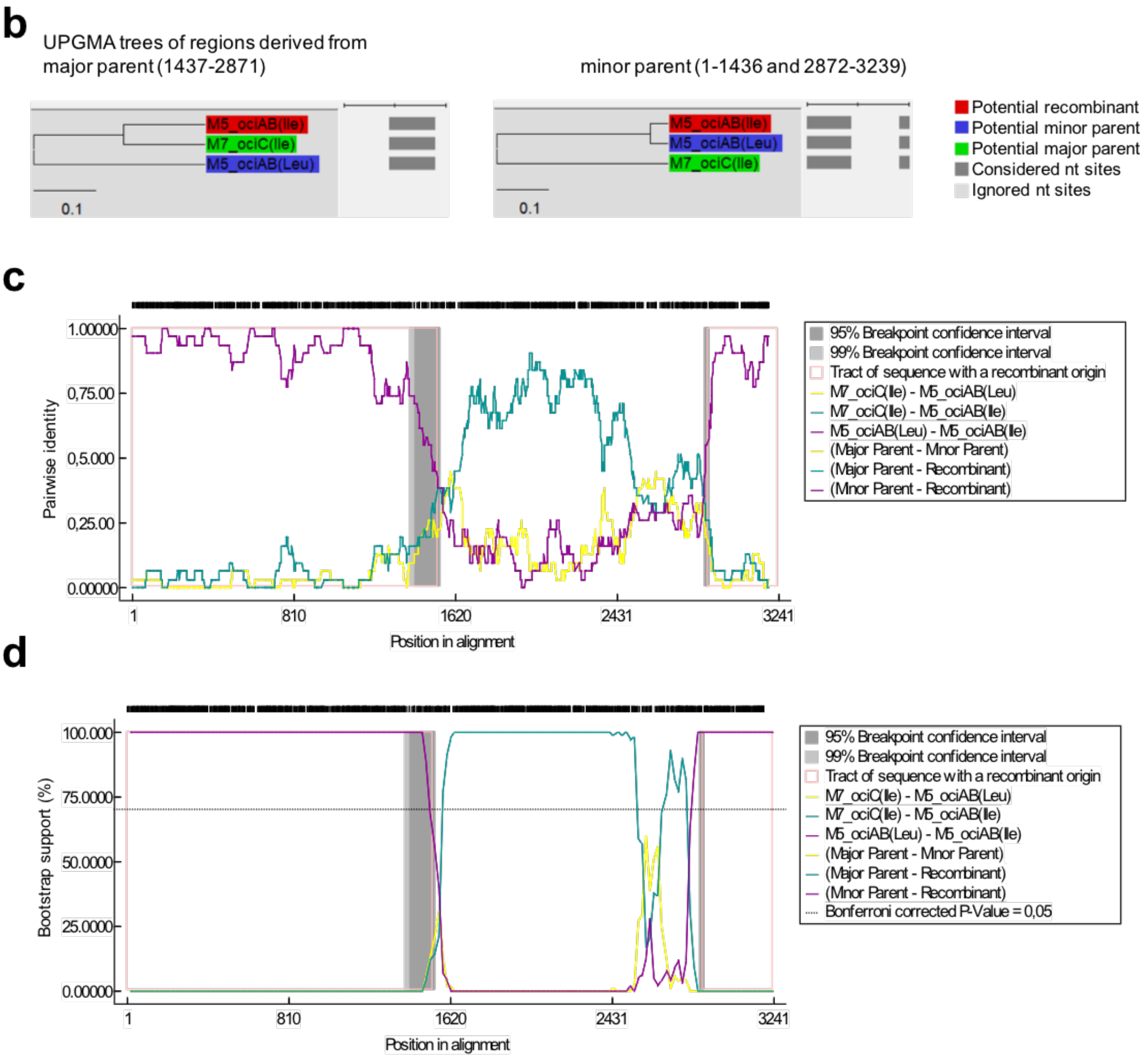

Supplementary FIG. S13. Statistical support for recombination event #13 by using Recombination Detection Program version 4 (RDP4) (Martin et al. 2015). **a**, Probability scores for recombination calculated by the recombination detection methods RDP (Martin and Rybicki 2000), GENECONV (Padidam et al. 1999), Bootscan (Salminen et al. 1995), Maxchi (Smith 1992), Chimaera (Posada and Crandall 2001), SiSscan (Gibbs et al. 2000), 3Seq (Boni et al. 2007) and LARD (Holmes et al. 1999) together with the prediction of the recombinant as well as the major and minor parent. RDP4 warns about a possible misidentification of the recombinant, which in this case (M5_ociAB(Ile)) seems logic. However, the assignment of M5_ociAB(Leu) as the minor parent does not make sense from a biosynthetic perspective. Therefore, we suggest M7_ociC(Ile) to be minor parent and M5_ociAB(Leu) to be the major parent. **b**, 'Recombination aware' phylogenetic trees of the putative recombination event. **c**, Graphical output of recombination detection method RDP. **d**, Graphical output of recombination detection method Bootscan.

a

14

			Breakpoint Positions in Recombinant					Detection Methods				
Recombinant	Minor Parent	Major Parent	Begin	End	RDP	GENECONV	Bootscan	Maxchi	Chimaera	SiSscan	3Seq	LARD
M6_ituC(Asn)	M6_mycC(Ser)	M7_ituC(Asn)	1181	27	4,47E-37	1,45E-26	9,30E-26	8,21E-27	1,16E-29	2,61E-39	4,10E-06	1,44E-84

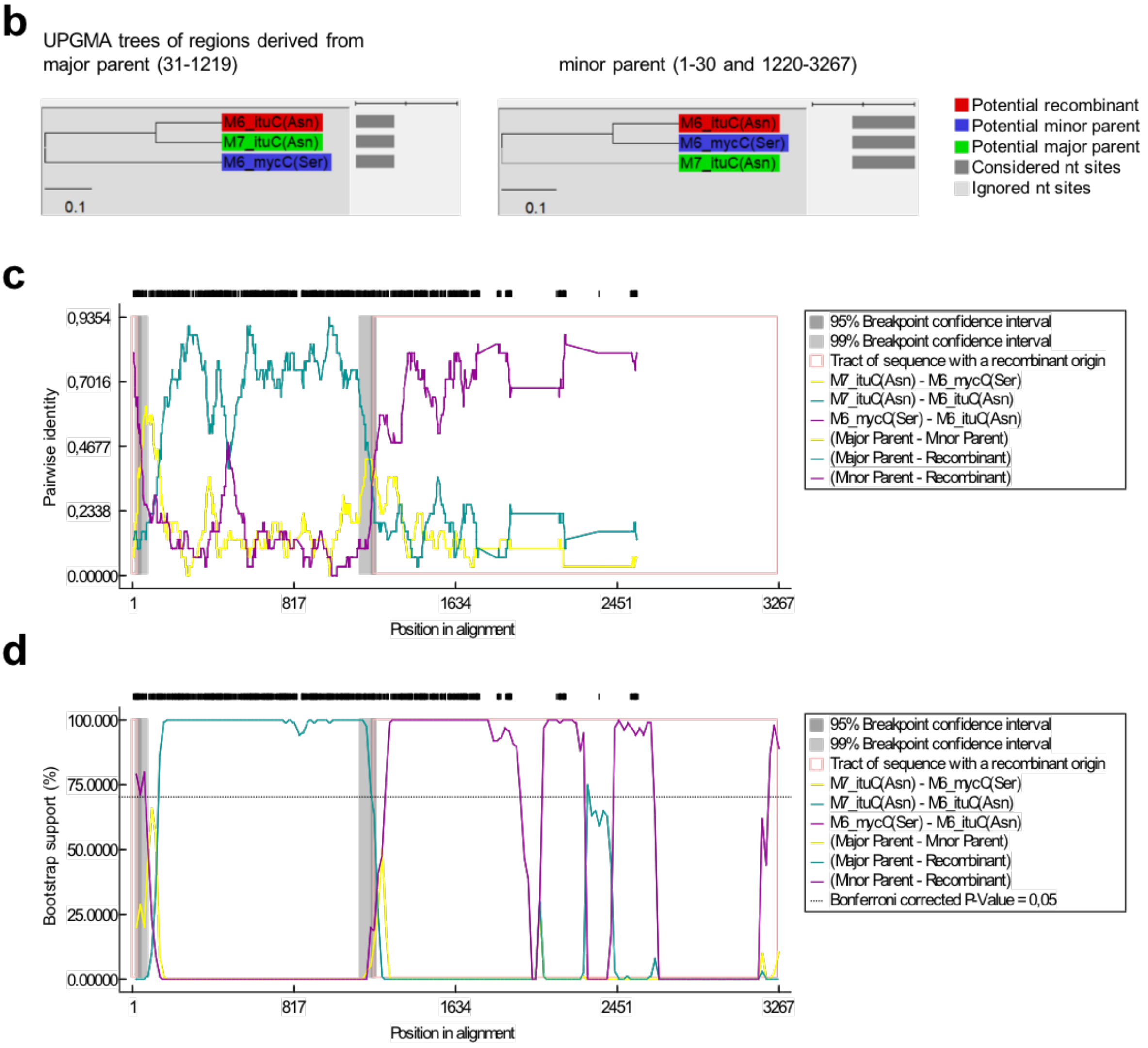

Supplementary FIG. S14. Statistical support for recombination event #14 by using Recombination Detection Program version 4 (RDP4) (Martin et al. 2015). **a**, Probability scores for recombination calculated by the recombination detection methods RDP (Martin and Rybicki 2000), GENECONV (Padidam et al. 1999), Bootscan (Salminen et al. 1995), Maxchi (Smith 1992), Chimaera (Posada and Crandall 2001), SiSscan (Gibbs et al. 2000), 3Seq (Boni et al. 2007) and LARD (Holmes et al. 1999) together with the prediction of the recombinant as well as the major and minor parent. RDP4 warns about a possible misidentification of the recombinant, which in this case (M6_ituC(Asn)) seems logic. However, the assignment of M6_mycC(Ser) as the minor parent does not make sense from a biosynthetic perspective. Therefore, we suggest M7_ituC(Asn) to be minor parent and M6_mycC(Ser) to be the major parent. **b**, ‘Recombination aware’ phylogenetic trees of the putative recombination event. **c**, Graphical output of recombination detection method RDP. **d**, Graphical output of recombination detection method Bootscan.

a

15

Recombinant	Minor Parent	Major Parent	Breakpoint Positions in Recombinant		Detection Methods							
			Begin	End	RDP	GENECONV	Bootscan	Maxchi	Chimaera	SiSscan	3Seq	LARD
M7_ituC(Ser)	M7_ituC(Asn)	M6_mycC(Ser)	2589	317	3,62E-72	7,18E-76	2,20E-70	7,23E-04	1,06E-15	1,08E-49	1,11E-16	7,68E-72

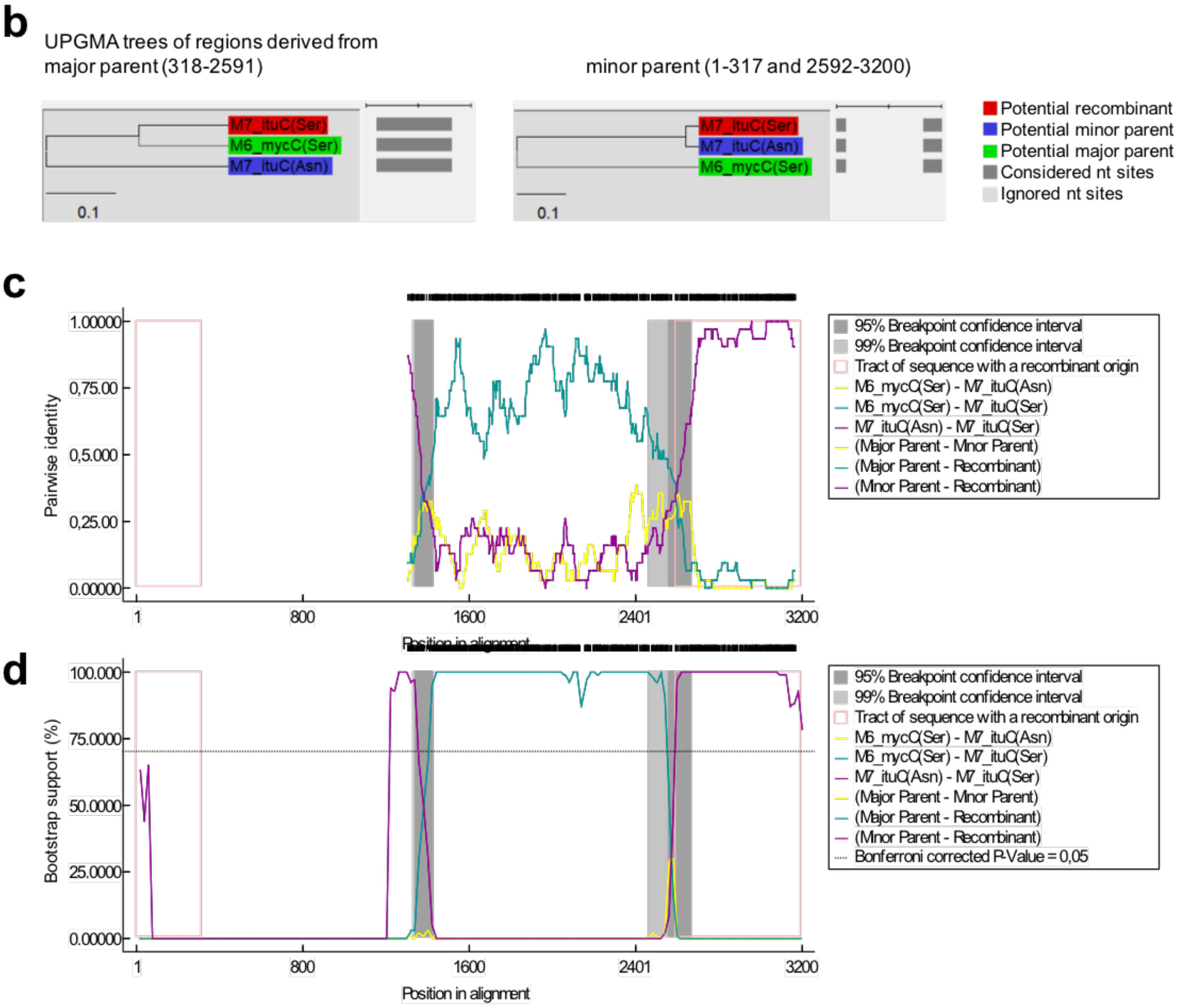

Supplementary FIG. S15. Statistical support for recombination event #15 by using Recombination Detection Program version 4 (RDP4) (Martin et al. 2015). **a**, Probability scores for recombination calculated by the recombination detection methods RDP (Martin and Rybicki 2000), GENECONV (Padidam et al. 1999), Bootscan (Salminen et al. 1995), Maxchi (Smith 1992), Chimaera (Posada and Crandall 2001), SiSscan (Gibbs et al. 2000), 3Seq (Boni et al. 2007) and LARD (Holmes et al. 1999) together with the prediction of the recombinant as well as the major and minor parent. RDP4 warns about a possible misidentification of the recombinant, which in this case (M7_ituC(Ser)) seems logic. However, the assignment of M7_ituC(Asn) as the minor parent does not make sense from a biosynthetic perspective. Therefore, we suggest M6_mycC(Ser) to be minor parent and M7_ituC(Asn) to be the major parent. **b**, 'Recombination aware' phylogenetic trees of the putative recombination event. **c**, Graphical output of recombination detection method RDP. **d**, Graphical output of recombination detection method Bootscan.

a

16

			Breakpoint Positions in Recombinant					Detection Methods				
Recombinant	Minor Parent	Major Parent	Begin	End	RDP	GENECONV	Bootscan	Maxchi	Chimaera	SiSscan	3Seq	LARD
M7_bmyC(Thr)	M1_fusA(Thr)	M7_ituC(Ser)	1836	2528	1,85E-50	3,15E-33	4,26E-39	1,56E-25	2,30E-09	8,40E-42	3,33E-16	5,82E-37

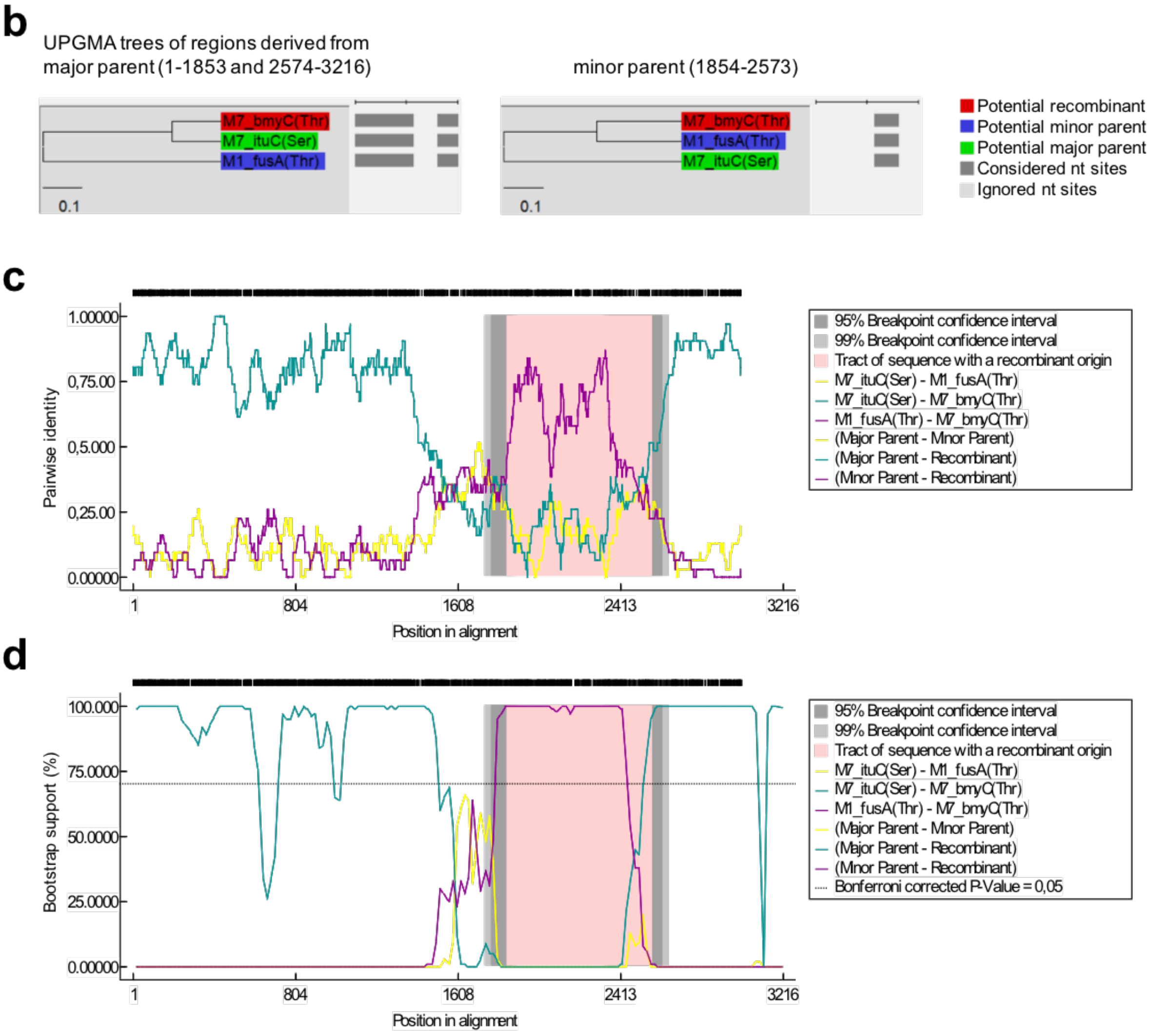

Supplementary FIG. S16. Statistical support for recombination event #16 by using Recombination Detection Program version 4 (RDP4) (Martin et al. 2015). **a**, Probability scores for recombination calculated by the recombination detection methods RDP (Martin and Rybicki 2000), GENECONV (Padidam et al. 1999), Bootscan (Salminen et al. 1995), Maxchi (Smith 1992), Chimaera (Posada and Crandall 2001), SiSscan (Gibbs et al. 2000), 3Seq (Boni et al. 2007) and LARD (Holmes et al. 1999) together with the prediction of the recombinant as well as the major and minor parent. RDP4 warns about a possible misidentification of the recombinant. However, the assignment seems logical. **b**, ‘Recombination aware’ phylogenetic trees of the putative recombination event. **c**, Graphical output of recombination detection method RDP. **d**, Graphical output of recombination detection method Bootscan.

a

(18)

			Breakpoint Positions in Recombinant		Detection Methods							
Recombinant	Minor Parent	Major Parent	Begin	End	RDP	GENECONV	Bootscan	Maxchi	Chimaera	SiSscan	3Seq	LARD
M7_pxmA(Thr)	M2_pxmA(Thr)	M7_pxmE(Leu)	1403	3007	1,42E-190	2,99E-167	1,26E-181	1,16E-66	8,40E-68	3,35E-83	2,22E-16	1,74E-82

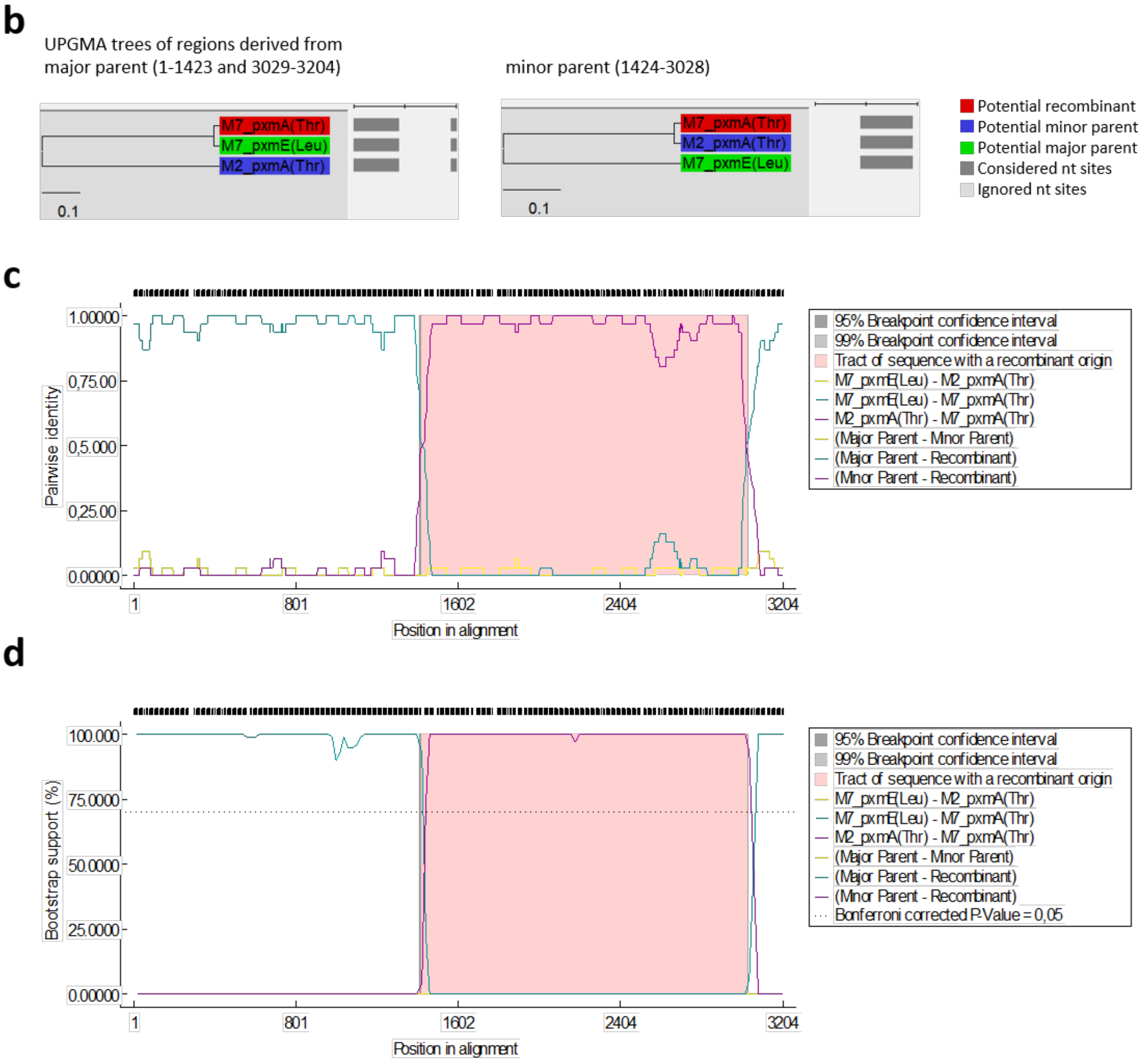

Supplementary FIG. S17. Statistical support for recombination event #18 by using Recombination Detection Program version 4 (RDP4) (Martin et al. 2015). **a**, Probability scores for recombination calculated by the recombination detection methods RDP (Martin and Rybicki 2000), GENECONV (Padidam et al. 1999), Bootscan (Salminen et al. 1995), Maxchi (Smith 1992), Chimaera (Posada and Crandall 2001), SiSscan (Gibbs et al. 2000), 3Seq (Boni et al. 2007) and LARD (Holmes et al. 1999) together with the prediction of the recombinant as well as the major and minor parent. RDP4 warns about a possible misidentification of the recombinant. However, the assignment seems logical. **b**, 'Recombination aware' phylogenetic trees of the putative recombination event. **c**, Graphical output of recombination detection method RDP. **d**, Graphical output of recombination detection method Bootscan.

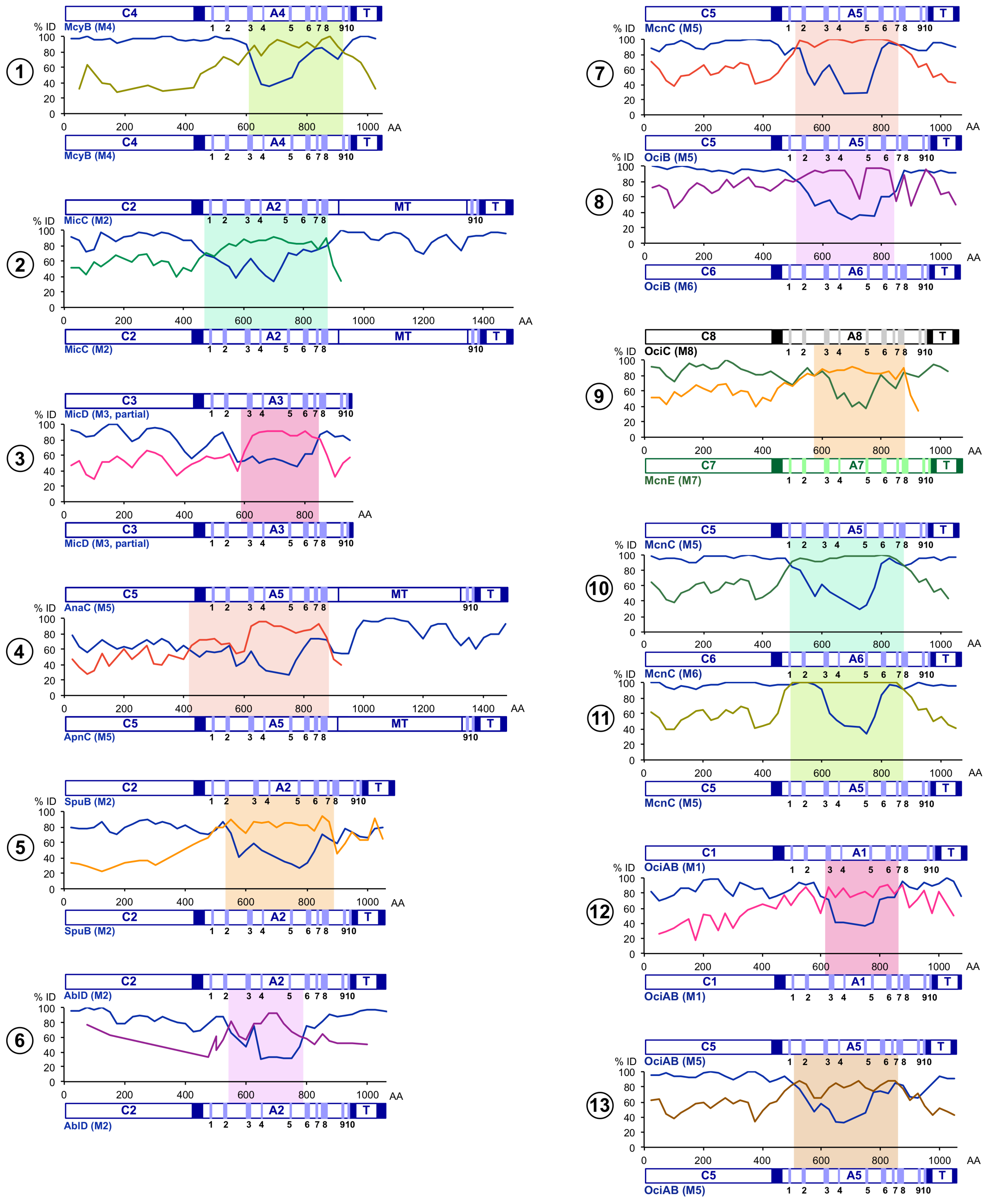

Supplementary FIG. S18. Subdomain exchange in cyanobacterial NRPS. Modules are divided in adenylation (A), condensation (C), thiolation (T), and if present methylation (MT) domains. Adenylation domain-specific core motifs are indicated by bands and numbers (1-10). Linkers are indicated as filled squares. Highlighted parts of the graphs represent regions that are more closely related to sequences encoding other modules than to sequence of the respective ortholog.

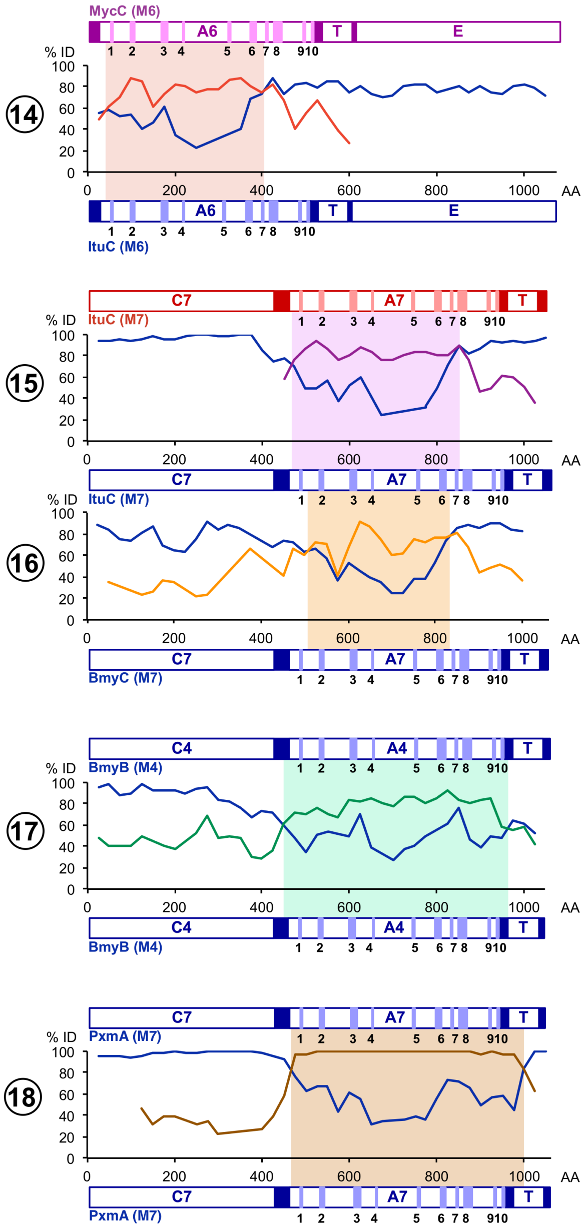

Supplementary FIG. S19. Subdomain exchange in non-cyanobacterial NRPS. Modules are divided in adenylation (A), condensation (C), thiolation (T) domains. Adenylation domain-specific core motifs are indicated by bands and numbers (1-10). Linkers are indicated as filled squares. Highlighted parts of the graphs represent regions that are more closely related to sequences encoding other modules than to sequence of the respective ortholog.

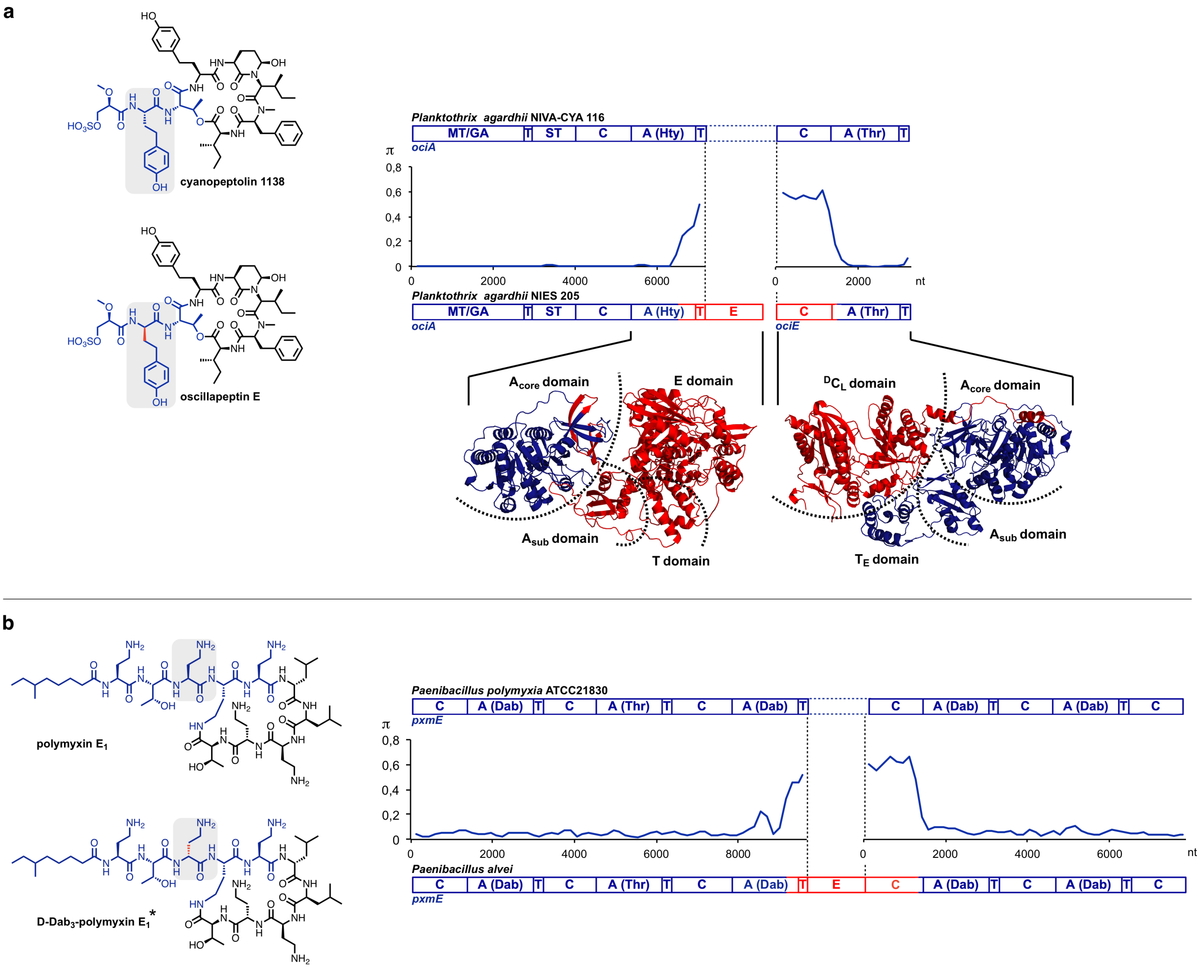

Supplementary FIG. S20. Integration of E domains in NRP assembly lines for the biosynthesis of **a**, cyanopeptolin 1138 and **b**, D-Dab$_3$-polymyxin E$_1$. The integration of of E domains is accompanied by the exchange of conventional T and $^{L}C_{L}$ domains with specialized T_E and $^{D}C_{L}$ domains associated with E domains. These results underpin the role of C domains as stereochemical gatekeepers and show that C domains are indeed exchanged if needed, thereby further undermining the attributed role of C domains as stringent selectivity filters during NRP biosynthesis. Gene segments encoding modules are divided in adenylation (A), condensation (C), thiolation (T), methyltransferases (MT), sulfotransferase (ST), and glyceric acid loading (SA) domains. In case of cyanopeptolin 1138 biosynthesis exchanged (sub)domains were illustrated in the NRPS (partial) structures of OciA and OciE, which have been modeled with Phyre2 (Kelley et al. 2015). Dab, diaminobutyric acid. *The stereochemistry of Dab in position 3 of D-Dab$_3$-polymyxin E$_1$ has not been validated experimentally. Instead a D-Dab residue in position 3 was assumed based on the presence of the epimerase domain (Tambadou et al. 2015). Therefore, the structural assignment is tentative. However, a similar pair with L- and D-Dab residues in position 3 has been reported for polymyxin B (Shaheen et al. 2011).

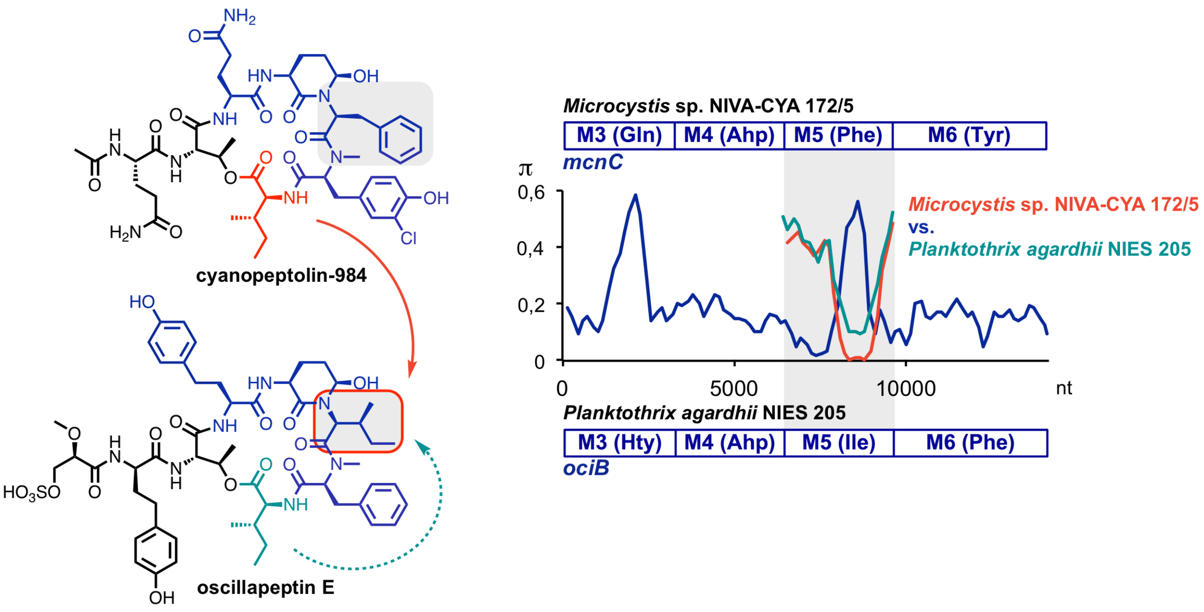

Supplementary FIG. S21. Evidence of recombination with horizontally acquired genes in the biosynthesis gene cluster of oscillapeptin E. There, parts of module 5 have a much more pronounced sequence similarity to module 7 of a distant relative than to the intra-cluster counterpart.

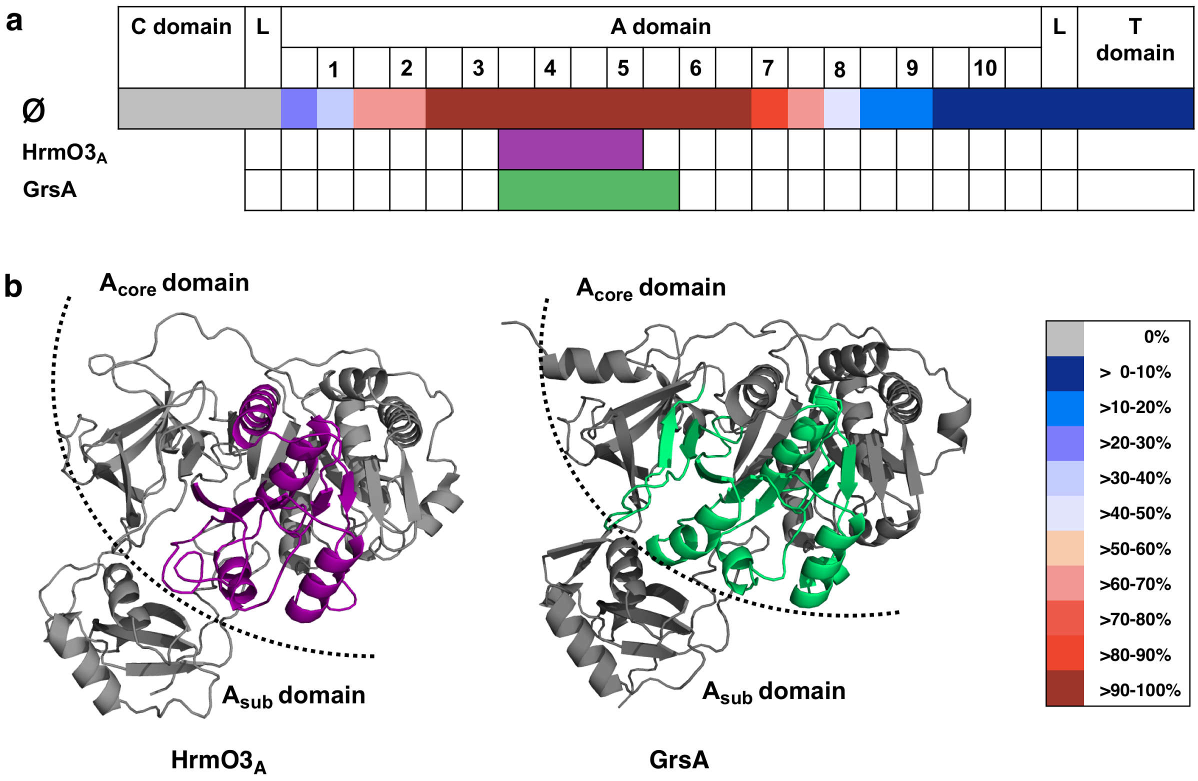

Supplementary FIG. S22. Natural exchange units vs. artificial subdomain swaps. **a**, Schematic comparison of natural exchange unit boundaries (as depicted in detail in **Fig. 5**) with artificial subdomain swaps in the engineering of $HrmO3_A$ (purple) and GrsA (green). **b**, Illustration of exchanged subdomains in the structure of $HrmO3_A$, which has been modeled with Phyre2 (Kelley et al. 2015) as well as in the crystal structure of GrsA (PDB ID: 1AMU) (Conti et al. 1997).

References

Bister B, Keller S, Baumann HI, Nicholson G, Weist S, Jung G, Süssmuth RD, Jüttner F. 2004. Cyanopeptolin 963A, a chymotrypsin inhibitor of *Microcystis* PCC 7806. *J Nat Prod.* 67:1755-1757.

Boni MF, Posada D, Feldman MW. 2007. An exact nonparametric method for inferring mosaic structure in sequence triplets. *Genetics* 176:1035-1047.

Choi SK, Park SY, Kim R, Kim SB, Lee CH, Kim JF, Park SH. 2009. Identification of a polymyxin synthetase gene cluster of *Paenibacillus polymyxa* and heterologous expression of the gene in *Bacillus subtilis*. *J Bacteriol.* 191:3350-3358.

Conti E, Stachelhaus T, Marahiel MA, Brick P. 1997. Structural basis for the activation of phenylalanine in the non-ribosomal biosynthesis of gramicidin S. *Embo J*. 16:4174-4183.

Duitman EH, Hamoen LW, Rembold M, Venema G, Seitz H, Saenger W, Bernhard F, Reinhardt R, Schmidt M, Ullrich C, et al. 1999. The mycosubtilin synthetase of *Bacillus subtilis* ATCC6633: a multifunctional hybrid between a peptide synthetase, an amino transferase, and a fatty acid synthase. *Proc Natl Acad Sci USA*. 96:13294-13299.

Dunlap CA, Bowman MJ, Rooney AP. 2019. Iturinic lipopeptide diversity in the *Bacillus subtilis* species group - Important antifungals for plant disease biocontrol applications. *Front Microbiol*. 10:1794.

Entfellner E, Frei M, Christiansen G, Deng L, Blom J, Kurmayer R. 2017. Evolution of anabaenopeptin peptide structural variability in the cyanobacterium *Planktothrix*. *Front Microbiol*. 8:219.

Fewer DP, Jokela J, Rouhiainen L, Wahlsten M, Koskenniemi K, Stal LJ, Sivonen K. 2009. The non-ribosomal assembly and frequent occurrence of the protease inhibitors spumigins in the bloom-forming cyanobacterium *Nodularia spumigena*. *Mol Microbiol.* 73:924-937.

Fewer DP, Osterholm J, Rouhiainen L, Jokela J, Wahlsten M, Sivonen K. 2011. Nostophycin biosynthesis is directed by a hybrid polyketide synthase-nonribosomal peptide synthetase in the toxic cyanobacterium *Nostoc* sp. strain 152. *Appl Environ Microbiol.* 77:8034-8040.

Fujii K, Sivonen K, Adachi K, Noguchi K, Sano H, Hirayama K, Suzuki M, Harada K. 1997. Comparative study of toxic and non-toxic cyanobacterial products: Novel peptides from toxic *Nodularia spumigena* AV1. *Tetrahedron Lett.* 38:5529–5532.

Fujii K, Sivonen K, Kashiwagi T, Hirayama K, K. H. 1999. Nostophycin, a novel cyclic peptide from the toxic cyanobacterium *Nostoc* sp. 152. *J Org Chem*. 64:5777-5782.

Gibbs MJ, Armstrong JS, Gibbs AJ. 2000. Sister-scanning: a Monte Carlo procedure for assessing signals in recombinant sequences. *Bioinformatics* 16:573-582.

Golakoti K, Yoshida WY, Chaganty S, Moore RE. 200. Isolation and structures of nostopeptolides A1, A2 and A3 from the cyanobacterium *Nostoc* sp. GSV224. *Tetrahedron* 56:9093-9102

Harada K, Fujii K, Shimada T, Suzuki M, Sano H, Adachi K, Carmichael WW. 1995. Two cyclic peptides, anabaenopeptins, a third group of bioactive compounds from the cyanobacterium *Anabaena flos-aquae* NRC 525-17. *Tetrahedron Lett.* 36:1511-1514.

Harada K, Nakano T, Fujii K, Shirai M. 2004. Comprehensive analysis system using liquid chromatography-mass spectrometry for the biosynthetic study of peptides produced by cyanobacteria. *J Chrom A*. 1033:107-113.

Höfer I, Crüsemann M, Radzom M, Geers B, Flachshaar D, Cai X, Zeeck A, Piel J. 2011. Insights into the biosynthesis of hormaomycin, an exceptionally complex bacterial signaling metabolite. *Chem Biol.* 18:381-391.

Hoffmann D, Hevel JM, Moore RE, Moore BS. 2003. Sequence analysis and biochemical characterization of the nostopeptolide A biosynthetic gene cluster from *Nostoc* sp. GSV224. *Gene* 311:171-180.

Holmes EC, Worobey M, Rambaut A. 1999. Phylogenetic evidence for recombination in dengue virus. *Mol Biol Evol*. 16:405-409.

Ishida K, Christiansen G, Yoshida WY, Kurmayer R, Welker M, Valls N, Bonjoch J, Hertweck C, Börner T, Hemscheidt T, et al. 2007. Biosynthesis and structure of aeruginoside 126A and 126B, cyanobacterial peptide glycosides bearing a 2-carboxy-6-hydroxyoctahydroindole moiety. *Chem Biol.* 14:565-576.

Ishida K, Matsuda H, Murakami M. 1998. Micropeptins 88-A to 88-F, chymotrypsin inhibitors from the cyanobacterium Microcystis aeruginosa (NIES-88). Tetrahedron 54:5545-5556.

Ishida K, Welker M, Christiansen G, Cadel-Six S, Bouchier C, Dittmann E, Hertweck C, Tandeau de Marsac N. 2009. Plasticity and evolution of aeruginosin biosynthesis in cyanobacteria. *Appl Environ Microbiol.* 75:2017-2026.

Itou Y, Ishida K, Shin HJ, Murakami M. 1999. Oscillapeptins A to F, serine protease inhibitors from the three strains of *Oscillatoria agardhii*. *Tetrahedron* 55:6871-6882.

Jokela J, Heinilä LMP, Shishido TK, Wahlsten M, Fewer DP, Fiore MF, Wang H, Haapaniemi E, Permi P, Sivonen K. 2017. Production of High Amounts of Hepatotoxin Nodularin and New Protease Inhibitors Pseudospumigins by the Brazilian Benthic *Nostoc* sp. CENA543. *Front Microbiol.* 8:1963.

Jokela J, Oftedal L, Herfindal L, Permi P, Wahlsten M, Døskeland SO, Sivonen K. 2012. Anabaenolysins, novel cytolytic lipopeptides from benthic *Anabaena* cyanobacteria. *PLoS One* 7:e41222.

Kajimura Y, Kaneda M. 1996. Fusaricidin A, a new depsipeptide antibiotic produced by *Bacillus polymyxa* KT-8. Taxonomy, fermentation, isolation, structure elucidation and biological activity. *J Antibiot.* 49:129-135.

Kaneko T, Nakajima N, Okamoto S, Suzuki I, Tanabe Y, Tamaoki M, Nakamura Y, Kasai F, Watanabe A, Kawashima K, et al. 2007. Complete genomic structure of the bloom-forming toxic cyanobacterium *Microcystis aeruginosa* NIES-843. *DNA Res.* 14:247-256.

Kautsar SA, Blin K, Shaw S, Navarro-Muñoz JC, Terlouw BR, van der Hooft JJJ, van Santen JA, Tracanna V, Suarez Duran HG, Pascal Andreu V, et al. 2020. MIBiG 2.0: a repository for biosynthetic gene clusters of known function. *Nucleic Acids Res.* 48:D454-d458.

Kelley LA, Mezulis S, Yates CM, Wass MN, Sternberg MJ. 2015. The Phyre2 web portal for protein modeling, prediction and analysis. *Nat Protoc.* 10:845-858.

Kimura Y, Murai E, Fujisawa M, Tatsuki T, Nobue F. 1969. Polymyxin P, new antibiotics of polymyxin group. *J Antibiot.* 22:449-450.

Koumoutsi A, Chen XH, Henne A, Liesegang H, Hitzeroth G, Franke P, Vater J, Borriss R. 2004. Structural and functional characterization of gene clusters directing nonribosomal synthesis of bioactive cyclic lipopeptides in *Bacillus amyloliquefaciens* strain FZB42. *J Bacteriol.* 186:1084-1096.

Kramer D. 2006. Microginin producing proteins and nucleic acids encoding a microginin gene cluster as well as methods for creating microginins. Patent: JP 2009517058-A.

Li J, Beatty PK, Shah S, Jensen SE. 2007. Use of PCR-targeted mutagenesis to disrupt production of fusaricidin-type antifungal antibiotics in *Paenibacillus polymyxa*. *Appl Environ Microbiol.* 73:3480-3489.

Ma Z, Wang N, Hu J, Wang S. 2012. Isolation and characterization of a new iturinic lipopeptide, mojavensin A produced by a marine-derived bacterium *Bacillus mojavensis* B0621A. *J Antibiot.* 65:317-322.

Martin D, Rybicki E. 2000. RDP: detection of recombination amongst aligned sequences. *Bioinformatics* 16:562-563.

Martin DP, Murrell B, Golden M, Khoosal A, Muhire B. 2015. RDP4: Detection and analysis of recombination patterns in virus genomes. *Virus Evol.* 1:vev003.

Matsuda H, Okino T, Murakami M, Yamaguchi K. 1996. Aeruginosins 102-A and B, new thrombin inhibitors from the cyanobacterium *Microcystis viridis* (NIES-102). *Tetrahedron* 52:14501-14506.

Matthiensen A, Beattie KA, Yunes JS, Kaya K, Codd GA. 2000. [D-Leu^{1}] Microcystin-LR, from the cyanobacterium *Microcystis* RST 9501 and from a *Microcystis* bloom in the Patos Lagoon estuary, Brazil. *Phytochemistry* 55:383-387.

Murakami M, Ishida K, Okino T, Okita Y, Matsuda H, Yamaguchi K. 1995. Aeruginosins 98-A and B, trypsin inhibitors from the blue-green alga *Microcystis aeruginosa* (NIES-98). *Tetrahedron Lett.* 36:2785-2788.

Neuhof T, Schmieder P, Preussel K, Dieckmann R, Pham H, Bartl F, von Döhren H. 2005. Hassallidin A, a glycosylated lipopeptide with antifungal activity from the cyanobacterium *Hassallia* sp. *J Nat Prod.* 68:695-700.

Nishizawa T, Ueda A, Nakano T, Nishizawa A, Miura T, Asayama M, Fujii K, Harada K, Shirai M. 2011. Characterization of the locus of genes encoding enzymes producing heptadepsipeptide micropeptin in the unicellular cyanobacterium *Microcystis*. *J Biochem.* 149:475-485.

Niu B, Vater J, Rueckert C, Blom J, Lehmann M, Ru JJ, Chen XH, Wang Q, Borriss R. 2013. Polymyxin P is the active principle in suppressing phytopathogenic *Erwinia* spp. by the biocontrol rhizobacterium *Paenibacillus polymyxa* M-1. *BMC Microbiol.* 13:137.

Okino T, Qi S, Matsuda H, Murakami M, Yamaguchi K. 1997. Nostopeptins A and B, elastase inhibitors from the cyanobacterium *Nostoc minutum. J Nat Prod.* 60:158-161.

Okumura HS, Philmus B, Portmann C, Hemscheidt TK. 2009. Homotyrosine-containing cyanopeptolins 880 and 960 and anabaenopeptins 908 and 915 from *Planktothrix agardhii* CYA 126/8. *J Nat Prod.* 72:172-176.

Padidam M, Sawyer S, Fauquet CM. 1999. Possible emergence of new geminiviruses by frequent recombination. *Virology* 265:218-225.

Park S-H, Kim JF, Lee C, Choi S-K, Jeong H, S-B. K. 2012. Polymyxin synthetase and gene cluster thereof. Patent: US 8329430 B2.

Pearson LA, Hisbergues M, Börner T, Dittmann E, Neilan BA. 2004. Inactivation of an ABC transporter gene, mcyH, results in loss of microcystin production in the cyanobacterium *Microcystis aeruginosa* PCC 7806. *Appl Environ Microbiol*. 70:6370-6378.

Peypoux F, Guinand M, Michel G, Delcambe L, Das BC, Lederer E. 1978. Structure of iturine A, a peptidolipid antibiotic from *Bacillus subtilis. Biochemistry* 17:3992-3996.

Peypoux F, Marion D, Maget-Dana R, Ptak M, Das BC, Michel G. 1985. Structure of bacillomycin F, a new peptidolipid antibiotic of the iturin group. *Eur J Biochem.* 153:335-340.

Peypoux F, Pommier MT, Das BC, Besson F, Delcambe L, Michel G. 1984. Structures of bacillomycin D and bacillomycin L peptidolipid antibiotics from *Bacillus subtilis*. J Antibiot. 37:1600-1604.

Peypoux F, Pommier MT, Marion D, Ptak M, Das BC, Michel G. 1986. Revised structure of mycosubtilin, a peptidolipid antibiotic from *Bacillus subtilis*. *J Antibiot.* 39:636-641.

Posada D, Crandall KA. 2001. Evaluation of methods for detecting recombination from DNA sequences: computer simulations. *Proc Natl Acad Sci USA* 98:13757-13762.

Rounge TB, Rohrlack T, Kristensen T, Jakobsen KS. 2008. Recombination and selectional forces in cyanopeptolin NRPS operons from highly similar, but geographically remote *Planktothrix* strains. *BMC Microbiol.* 8:141.

Rounge TB, Rohrlack T, Nederbragt AJ, Kristensen T, Jakobsen KS. 2009. A genome-wide analysis of nonribosomal peptide synthetase gene clusters and their peptides in a *Planktothrix rubescens* strain. *BMC Genom*. 10:396.

Rounge TB, Rohrlack T, Tooming-Klunderud A, Kristensen T, Jakobsen KS. 2007. Comparison of cyanopeptolin genes in Planktothrix, Microcystis, and *Anabaena* strains: evidence for independent evolution within each genus. *Appl Environ Microbiol.* 73:7322-7330.

Salminen MO, Carr JK, Burke DS, McCutchan FE. 1995. Identification of breakpoints in intergenotypic recombinants of HIV type 1 by bootscanning. *AIDS Res Hum Retroviruses* 11:1423-1425.

Sano T, Kaya K. 1997. A 3-amino-10-chloro-2-hydroxydecanoic acid-containing tetrapeptide from *Oscillatoria agardhii. Phytochemistry* 44:1503-1505.

Sano T, Kaya K. 1996. Oscillapeptin G, a tyrosinase inhibitor from toxic *Oscillatoria agardhii. J Nat Prod.* 59:90-92.

Shaheen M, Li J, Ross AC, Vederas JC, Jensen SE. 2011. *Paenibacillus polymyxa* PKB1 produces variants of polymyxin B-type antibiotics. *Chem Biol.* 18:1640-1648.

Shishido TK, Jokela J, Kolehmainen CT, Fewer DP, Wahlsten M, Wang H, Rouhiainen L, Rizzi E, De Bellis G, Permi P, et al. 2015. Antifungal activity improved by coproduction of

cyclodextrins and anabaenolysins in Cyanobacteria. *Proc Natl Acad Sci USA.* 112:13669-13674.
Shishido TK, Kaasalainen U, Fewer DP, Rouhiainen L, Jokela J, Wahlsten M, Fiore MF, Yunes JS, Rikkinen J, Sivonen K. 2013. Convergent evolution of [D-Leucine[1]] microcystin-LR in taxonomically disparate cyanobacteria. *BMC Evol Biol.* 13:86.
Sivonen K, Namikoshi M, Evans WR, Färdig M, Carmichael WW, Rinehart KL. 1992. Three new microcystins, cyclic heptapeptide hepatotoxins, from *Nostoc* sp. strain 152. *Chem Res Toxicol.* 5:464-469.
Smith JM. 1992. Analyzing the mosaic structure of genes. *J Mol Evol.* 34:126-129.
Sun Q, Ishida K, Matsuda H, Murakami M. 1998. Enzyme inhibitors from *Nostoc. Symp Chem Nat Prod.* 40:499-504.
Tambadou F, Caradec T, Gagez AL, Bonnet A, Sopéna V, Bridiau N, Thiéry V, Didelot S, Barthélémy C, Chevrot R. 2015. Characterization of the colistin (polymyxin E1 and E2) biosynthetic gene cluster. *Arch Microbiol.* 197:521-532.
Thaker MN, Wang W, Spanogiannopoulos P, Waglechner N, King AM, Medina R, Wright GD. 2013. Identifying producers of antibacterial compounds by screening for antibiotic resistance. *Nat Biotechnol.* 31:922-927.
Tooming-Klunderud A, Rohrlack T, Shalchian-Tabrizi K, Kristensen T, Jakobsen KS. 2007. Structural analysis of a non-ribosomal halogenated cyclic peptide and its putative operon from Microcystis: implications for evolution of cyanopeptolins. *Microbiology* 153:1382-1393.
Tsuge K, Akiyama T, Shoda M. 2001. Cloning, sequencing, and characterization of the iturin A operon. *J Bacteriol.* 183:6265-6273.
Vanittanakom N, Loeffler W, Koch U, Jung G. 1986. Fengycin--a novel antifungal lipopeptide antibiotic produced by *Bacillus subtilis* F-29-3. *J Antibiot.* 39:888-901.
Vestola J, Shishido TK, Jokela J, Fewer DP, Aitio O, Permi P, Wahlsten M, Wang H, Rouhiainen L, Sivonen K. 2014. Hassallidins, antifungal glycolipopeptides, are widespread among cyanobacteria and are the end-product of a nonribosomal pathway. *Proc Natl Acad Sci. USA.* 111:E1909-1917.
Volpon L, Tsan P, Majer Z, Vass E, Hollósi M, Noguéra V, Lancelin JM, Besson F. 2007. NMR structure determination of a synthetic analogue of bacillomycin Lc reveals the strategic role of L-Asn1 in the natural iturinic antibiotics. *Spectrochim Acta A.* 67:1374-1381.
Wilkinson S, Lowe LA. 1964. Structures of polymycin B2 and polymycin E1. *Nature* 204:993-994.
Wilkinson S, Lowe LA. 1966. Structures of the polymyxins A and the question of identity with the polymyxins M. *Nature* 212:311.

Williams DH, Kalman J. 1977. Structural and mode of action studies on the antibiotic vancomycin. Evidence from 270-MHz proton magnetic resonance. *J Am Chem Soc.* 99:2768-2774.

Xu L, Huang H, Wei W, Zhong Y, Tang B, Yuan H, Zhu L, Huang W, Ge M, Yang S, et al. 2014. Complete genome sequence and comparative genomic analyses of the vancomycin-producing Amycolatopsis orientalis. *BMC Genom.* 15:363.

Zlatopolskiy BD, Loscha K, Alvermann P, Kozhushkov SI, Nikolaev SV, Zeeck A, de Meijere A. 2004. Final elucidation of the absolute configuration of the signal metabolite hormaomycin. *Chemistry* 10:4708-4717.
